# Assessing rates of parasite coinfection and spatiotemporal strain variation via metabarcoding: Insights for the conservation of European turtle doves *Streptopelia turtur*

**DOI:** 10.1111/mec.16421

**Published:** 2022-04-02

**Authors:** Rebecca C. Thomas, Jenny C. Dunn, Deborah A. Dawson, Helen Hipperson, Gavin J. Horsburgh, Antony J. Morris, Chris Orsman, John Mallord, Philip V. Grice, Keith C. Hamer, Cyril Eraud, Lormée Hervé, Simon J. Goodman

**Affiliations:** ^1^ 4468 School of Biology University of Leeds Leeds UK; ^2^ 7315 Department of Animal and Plant Sciences NERC Biomolecular Analysis Facility University of Sheffield Sheffield UK; ^3^ RSPB Centre for Conservation Science Royal Society for the Protection of Birds The Lodge Sandy Bedfordshire UK; ^4^ Department of Life Sciences Joseph Banks Laboratories University of Lincoln Lincoln UK; ^5^ Natural England Peterborough UK; ^6^ Office Français de la Biodiversité Direction de la Recherche et de l'Appui Scientifique Villiers‐en‐Bois France

**Keywords:** coinfection, haemosporidians, high‐throughput sequencing, next‐generation sequencing, NGS, *Trichomonas gallinae*

## Abstract

Understanding the frequency, spatiotemporal dynamics and impacts of parasite coinfections is fundamental to developing control measures and predicting disease impacts. The European turtle dove (*Streptopelia turtur*) is one of Europe's most threatened bird species. High prevalence of infection by the protozoan parasite *Trichomonas gallinae* has previously been identified, but the role of this and other coinfecting parasites in turtle dove declines remains unclear. Using a high‐throughput sequencing approach, we identified seven strains of *T*. *gallinae*, including two novel strains, from ITS1/5.8S/ITS2 ribosomal sequences in turtle doves on breeding and wintering grounds, with further intrastrain variation and four novel subtypes revealed by the iron‐hydrogenase gene. High spatiotemporal turnover was observed in *T*. *gallinae* strain composition, and infection was prevalent in all populations (89%–100%). Coinfection by multiple *Trichomonas* strains was rarer than expected (1% observed compared to 38.6% expected), suggesting either within‐host competition, or high mortality of coinfected individuals. In contrast, coinfection by multiple haemosporidians was common (43%), as was coinfection by haemosporidians and *T*. *gallinae* (90%), with positive associations between strains of *T*. *gallinae* and *Leucocytozoon* suggesting a mechanism such as parasite‐induced immune modulation. We found no evidence for negative associations between coinfections and host body condition. We suggest that longitudinal studies involving the recapture and investigation of infection status of individuals over their lifespan are crucial to understand the epidemiology of coinfections in natural populations.

## 1 INTRODUCTION

Coinfection, defined as the simultaneous infection of an individual host by multiple parasite species or strains, and also known as multiple infection or polyparasitism, is more common than single infections in natural populations (Telfer et al., 2010; Thumbi et al., 2014). The dynamics of coinfections may differ significantly from those expected for single parasite infections, due to potential conflicts between parasites, both in virulence and transmission strategies (Davies et al., 2002; Gower & Webster, 2005; Nowak & May, 1994; Schjørring & Koella, 2003; van Baalen & Sabelis, 1995). Despite this, the majority of research on parasite epidemiology in wild populations tends to focus on single host‐parasite interactions (Bordes & Morand, 2011; Pedersen et al., 2007; Petney & Andrews, 1998; Rigaud et al., 2010). In this study, we examine spatiotemporal variation in coinfecting *Trichomonas gallinae* lineages across multiple populations of endangered European turtle doves *Streptopelia turtur* (hereafter turtle doves), and evaluate the frequency of coinfections by multiple haemosporidians in breeding populations, to gain new epidemiological insights into parasite coinfections relevant to conservation management.

Coinfections result from the sequential or simultaneous exposure of hosts to multiple parasites; this can result in interactions between parasite strains that are neutral, facilitative, or antagonistic (Karvonen et al., 2019), with implications for both parasite and host fitness (Johnson & Hoverman, 2012). The likelihood of an individual being coinfected by multiple parasites, or by multiple strains of the same parasite, is dependent on both the likelihood of exposure, and the diversity of parasites, or parasite strains, circulating within the host or vector population or in the environment, and may vary spatiotemporally (e.g., Brooker & Clements, 2009). The outcome of coinfections for the host depends on interactions between these multiple parasite strains (e.g., Kinnula et al., 2017). First, there may be antagonistic interactions between the different strains, resulting in one strain outcompeting the other within the host and a lower than expected occurrence of coinfections overall, although this pattern may also be driven through increased host mortality (Clay et al., 2019; Palinauskas et al., 2011). Second, parasite interactions may be facultative, with each parasite benefiting from the presence of the other (Clerc et al., 2019), potentially resulting in a higher than expected prevalence of coinfection, especially between certain parasites or strains (Clay et al., 2019). Finally, parasites may not interact within a host, or interactions between parasites may be neutral, in which case coinfections should occur at a rate representative of parasite prevalence in the wider population. Therefore, observing patterns of coinfection within host populations can provide insights into the mechanisms driving these patterns.

Sequencing using parallel metabarcoding approaches has facilitated the jump from identifying single organisms to whole communities simultaneously (Cristescu, 2014). The literature on microbial and viral biodiversity, and interactions with ecological and disease processes has grown rapidly in recent years (e.g., Pagenkopp Lohan et al., 2016; Pompanon et al., 2012; Tedersoo et al., 2014; Wu et al., 2016). However, only relatively few studies have targeted parasites, including studies profiling the intestinal nematode communities of rufous mouse lemurs *Microcebus rufus* (Aivelo et al., 2015), identifying the causative agent and vector involved in a Sindbis virus outbreak in Sweden (Bergqvist et al., 2015); characterisation of black band disease on coral *Porites lutea* (Séré et al., 2016); and the simultaneous assessment of gut parasites in addition to diet in the banded leaf monkey *Presbytis femoralis* (Srivathsan et al., 2016). It is important to consider disease as a potential driver in species declines (Daszak et al., 2000; Heard et al., 2013; Stockdale et al., 2015; Tompkins & Jakob‐Hoff, 2011) and metabarcoding is rapidly becoming an essential tool for the fields of disease ecology and conservation.

Turtle doves breed across Europe and into central Asia, with the entire population overwintering in sub‐Saharan Africa. Across much of the range, populations are undergoing severe declines and the conservation status is considered Vulnerable on a global scale (Birdlife International, 2017). Turtle doves are on the red list of birds of conservation concern in the UK and are also the UK’s fastest declining breeding bird (Hayhow et al., 2017). Possible drivers behind their population decline include a decrease in breeding productivity driven by a reduction in food availability on the breeding grounds (Browne & Aebischer, 2003, 2004; Dunn, Morris & Grice, 2017), hunting during migration (Boutin, 2001), degradation of overwintering habitat (Tucker & Heath, 1994) and variable food availability whilst overwintering (Eraud et al., 2009).

UK breeding turtle doves have a high prevalence of infection by *Trichomonas gallinae* (Lennon et al., 2013; Stockdale et al., 2015), and by haemosporidian parasites (*Haemoproteus*, *Plasmodium* or *Leucocytozoon*; Dunn, Stockdale et al., 2017). In columbids, *T*. *gallinae* transmission is generally from parent to offspring via regurgitated crop milk, but may also occur via direct contact between infected and uninfected individuals whilst feeding, during courtship (Kocan & Herman, 1971; Stabler, 1954), or via indirect inter‐ or intraspecific transmission at food and water sources (Anderson et al., 2009; Lawson et al., 2012). Infection with *T*. *gallinae* can cause sporadic outbreaks of disease (trichomonosis) resulting in population crashes in Columbiformes (Girard et al., 2014; Höfle et al., 2004; Villanúa et al., 2006); the parasite is responsible for decreased survival rates in adults of the endangered Mauritian pink pigeon *Columba mayeri* and is a major mortality factor in squabs and fledglings (Bunbury et al., 2007, 2008). Furthermore, infection by *T*. *gallinae* has been linked to mortality in both adult and nestling turtle doves (Stockdale et al., 2015). Haemosporidian parasites are vector‐transmitted and may be transmitted on breeding or wintering grounds for migratory species (Hasselquist et al., 2007) such as the turtle dove. Infections tend to be subclinical, but can depress breeding productivity and/or survival (Knowles et al., 2010; Lachish et al., 2011). Coinfections by *T*. *gallinae* and the haemosporidian *Leucocytozoon marchouxi* in breeding Pink pigeons result in the failure of chicks to fledge (Bunbury, 2006). Consequently, coinfections of *T*. *gallinae* and haemosporidian parasites in turtle doves, as well as the potential presence of more than one strain of each parasite group, may be cause for concern.

Previous studies reporting prevalence and genetic diversity of *T*. *gallinae* infection in wild bird populations have relied on Sanger sequencing to identify genetic strains based on the ITS1/5.8S/ITS2 ribosomal region (hereafter referred to as the ITS region) and the iron hydrogenase gene (hereafter referred to as the *Fe*‐*hyd* region; Chi et al., 2013; Gerhold et al., 2008; Martínez‐Herrero et al., 2014). Only one prior study has investigated coinfection between *T*. *gallinae* strains through cloning and culturing, which revealed that two pigeons out of 17 sampled were coinfected by two strains (Grabensteiner et al., 2010). Coinfecting haemosporidian strains are difficult to detect as the universal primers generally used to detect infections (Hellgren et al., 2004; Waldenström et al., 2004) coamplify multiple strains where present, meaning that Sanger sequencing can be unreliable (Bernotienė et al., 2016). Currently, recommended methods of detecting coinfections include microscopic examination of samples (Valkiūnas et al., 2006), which is time consuming and can still miss low intensity infections, or lineage specific qPCR (Asghar et al., 2011), which requires prior knowledge of the lineages present within hosts.

In this study, we developed high throughout sequencing (HTS) methods to examine the spatiotemporal dynamics of parasite coinfections, validated these methods using multiple host populations across breeding and wintering grounds, and examined the potential implications of coinfection. As genetic evidence suggests that turtle dove populations are panmictic (Calderón et al., 2016), we predicted that little variation in parasite strain composition would be present across spatially distinct populations, but that temporal variation may be evident, although our spatiotemporal sampling was necessarily confounded by the migratory nature of the species. Previous studies have suggested that the prevalence of *T*. *gallinae* infection, and haemoparasite infection, is high (Dunn, Stockdale, et al., 2017; Lennon et al., 2013; Stockdale et al., 2015), and thus we predicted high rates of coinfection, both within and between *T*. *gallinae* and haemosporidians. We then tested whether coinfections occur at random, or whether positive or negative associations are present between different parasite strains, and finally we examined potential impacts of coinfection on body condition. Together these provide novel insights into rates of coinfection, geographic strain distribution and temporal turnover of parasite lineages on both breeding and wintering grounds of a rapidly declining bird, with implications for the management and conservation of turtle dove populations.

## 2 MATERIALS AND METHODS

### 2.1 Sites and sample collection

Breeding turtle doves in the UK were captured using whoosh nets (Redfern & Clark, 2001) at temporary bait sites between May and July 2013–2015 on seven farms in Essex, four farms in Norfolk/Cambridgeshire (detailed in Dunn et al., 2018) and three additional farms in Hampshire (50°58′N, 01°55′W; 50°58′N, 01°55′W and 50°57′N, 01°55′W). Samples to screen for *T*. *gallinae* were taken from the mouth cavity, oesophagus and crop using a moistened sterile viscose swab. Swabs were inoculated into individual InPouch TF culture kits (Biomed Diagnostics), sealed and incubated at 37°C for 3–7 days in order to culture *T*. *gallinae* parasites (Bunbury et al., 2005). All birds were ringed using standard British Trust for Ornithology (BTO) metal rings, and had a blood sample taken to screen for the presence of haemosporidians, which was stored either at room temperature on Whatman FTA cards (GE Healthcare Life Sciences), or frozen at −20°C within 1–8 h. Previously published *T*. *gallinae* data from 2011 to 2012, with samples collected from the same sites using the same methods (Lennon et al., 2013; Stockdale et al., 2015), were also included in subsequent analyses.

Sample collection at breeding sites in western France was undertaken at two locations: Chizé Forest (46°6′N, 0°21′W) and Oléron Island (45°55′N, 01°16′W). Birds were caught using baited potter traps during a two week period (24 May–7 June 2014) and sampled for *T*. *gallinae* as above.

Using a combination of whoosh and mist nets, over‐wintering turtle doves were caught in Oursi, Burkina Faso (14°41′N, 0°27′W) from November 2012 to April 2013, and at a site near Sandiara, Senegal (14°24′N, 16°47′W) from January to March in 2014 and 2015, and sampled as above. Issues with export permits led to samples from Burkina Faso being stored in refrigerated conditions (4°C) for approximately 18 months before being imported. Storage at −20°C is recommended, so some sample degradation is likely. *Trichomonas gallinae* samples collected in Senegal in 2014 were immediately isolated from the media after incubation, as described below. Each *T*. *gallinae* sample collected in Senegal in 2015 was split, and half stored with equal amounts of ethanol in an Eppendorf and half on Whatman FTA Classic cards (GE Healthcare Life Sciences), as part of a separate project and as described in Thomas et al. (2022), before being imported to the UK where parasites were isolated from the media.

### 2.2 *Trichomonas gallinae* isolation

For all samples, *T*. *gallinae* were isolated following the protocol of Riley et al. (1992), modified as follows: 2.5 ml of culture was centrifuged at 2100 *g* for 5 min, the resulting pellet was washed with 1 ml of phosphate‐buffered saline (PBS) by centrifugation and then resuspended in 200 µl of PBS. Samples were then stored at −20°C before DNA extraction.

### 2.3 DNA extraction and PCR

DNA extraction from UK parasite samples collected in 2013, and from blood samples, was carried out using a DNeasy blood and tissue kit (Qiagen). DNA was extracted from all other *T*. *gallinae* samples using a modified ammonium acetate method (Nicholls et al., 2000). Briefly, the parasite pellet was digested overnight in digestion buffer (20 mM EDTA, 50 mM Tris, 120 mM NaCl, 1% SDS, pH 8.0) with 50 µg of Proteinase K (Sigma‐Aldrich). Ammonium acetate (4 M) was then used to precipitate out the proteins and ethanol precipitated out the DNA. The resulting DNA pellet was dissolved in 20–50 µl low TE buffer (10 mM Tris‐HCl, 0.1 mM EDTA, pH 8.0), depending on the size of the pellet, in a water bath at 65°C. Extracted DNA was stored at −20°C. Samples were not individually quantified but based on a subset of extractions they typically ranged from 0.5 to 60 ng/µl.

PCR reactions were run on either a GeneAmp 9700 PCR system (Applied Biosystems) or a DNA Engine Tetrad 2 (Bio‐Rad Laboratories Inc), and a negative control of molecular grade water and a positive control of known *T*. *gallinae* DNA were included in each PCR run. PCR protocols are detailed in Table 1, and primer sequences and expected product lengths are given in Table 2. PCR products were electrophoresed through a 1%–1.5% agarose gel, stained either with GelRed (Biotium) or ethidium bromide, in 1 × TBE buffer and visualised by UV light. The presence of an amplicon band at the expected product size (Table 2) indicated the presence of infection.

**TABLE 1:** PCR thermocycler conditions used for each primer set according to sample origin

Primers	Target	Samples	PCR conditions					
			Initial denaturation	Cycle number	Denaturation	Annealing	Extension	Final extension
TFR1, TFR2	*Trichomonas* ITS	UK 2013	5 min/94°C	35	45 s/94°C	30 s/63°C	45 s/72°C	5 min/72°C
TFR1, TFR2	*Trichomonas* ITS	All but UK 2013	15 min/95°C	11	60 s/94°C	30 s/66°C–56°C (1°C decrease per cycle)	60 s/72°C	
				Then 24	60 s/94°C	30 s/55°C	60 s/72°C	10 min/72°C
TrichhydFor TrichhydREV	*Trichomonas Fe‐hyd*	UK 2013	5 min/94°C	35	45 s/94°C	30 s/53°C	45 s/72°C	5 min/72°C
FeH1FOR‐REV FeH2FOR‐REV FeH3FOR‐REV FeH4FOR‐REV	*Trichomonas Fe‐hyd*	All but UK 2013	5 min/94°C	35	45 s/94°C	30 s/53°C	45 s/72°C	5 min/72°C
HMRf, H15730	Haemoparasite	All UK	15 min/95°C	35	30 s/94°C	60 s/52°C	90 s/72°C	10 min/72°C
LeuNew 1F, LDRd	Haemoparasite	All UK	15 min/95°C	35	30 s/95°C	60 s/56°C	60 s/72°C	10 min/72°C

**TABLE 2:** Primer sets and sequences for amplification of the ITS region (TFR1; TFR2) and the *Fe*‐*hyd* region (Trichhyd; FeH1–FeH4) from *Trichomonas gallinae* and the cytochrome *b* region of the mitochondrial genome from haemosporidia (HMRf; H15730; Leunew1F; LDRd), along with product length and original source

Forward primer	Forward primer sequence (5′−3′)	Reverse primer	Reverse primer sequence (5′−3′)	Product length (bp)	Citation
TFR1	TGCTTCAGTTCAGCGGGTCTTCC	TFR2	CGGTAGGTGAACCTGCCGTTGG	400	Gaspar da Silva et al. (2007)
TrichhydFOR	GTTTGGGATGGCCTCAGAAT	TrichhydREV	AGCCGAAGATGTTGTCGAAT	1000	Lawson et al. (2011)
FeH1FOR	GCCACGATGAAACATGCTC	FeH1REV	ACCGACTGGGCAATAGAGTG	326	This study
FeH2FOR	CACATCCGCCATCATCTTC	FeH2REV	GCAGATTGTAAGGTCAGCA	349	This study
FeH3FOR	TTGGCTACAAGGAGGGTACAG	FeH3REV	CGAGGAGCTTTGGAAGGTAG	302	This study
FeH4FOR	TTGGGTTAACTACGTTGAGCAG	FeH4REV	GAAGCCGAAGATGTTGTCG	325	This study
HMRf	GGTAGCWCTAATCCTTTAGG	H15730	CATCCAATCCATAATAAAGCAT	378	Fallon et al. (2003), Martínez et al. (2009)
Leunew1F	GGWCAAATGAGTTTCTGGG	LDRd	CTGGATGWGATAATGGWGCA	302	Merino et al. (2008), Quillfeldt et al. (2014)

### 2.4 *Trichomonas gallinae* ITS 1/5.8S/ITS 2 ribosomal region

A 400 bp length of the ITS ribosomal region was targeted using primers TFR1 and TFR2 (Table 2). Samples from 2013 were amplified in a 50 µl reaction volume comprising 1× PCR buffer (Promega), 2 mM MgCl₂, 0.2 mM dNTP mix (Promega), 0.5 µM forward and reverse primer, 1.25 U of GoTaq Hot Start *Taq* DNA Polymerase (Promega) and 1 µl of DNA. All other samples were amplified in a 10 µl reaction volume comprising 0.8× Qiagen Multiplex PCR Master Mix (Qiagen), 0.5 µM forward and reverse primer and 1 µl of DNA.

### 2.5 *Trichomonas gallinae* Fe‐hydrogenase region

The full 1000 bp length of the *Fe*‐*hyd* gene region was targeted using the primers TrichhydFOR and TrichhydREV (Table 2). Samples from 2013 were amplified as per TFR1 and TFR2, but with 3 mM MgCI₂, 0.25 µM dNTP mix, 0.25 µM forward and reverse primer and 5 U Go Taq Hot Start *Taq* DNA Polymerase in a 50 μl reaction volume.

To allow HTS using an Illumina MiSeq, new primer sets were designed to amplify the 1000 bp *Fe*‐*hyd* region in four overlapping sections 300–350 bp in length (Table 2). These primer sets were designed using Primer 3 v.0.4.0 (Koressaar & Remm, 2007; Untergasser et al., 2012) based on a consensus sequence of all the available *Fe*‐*hyd* sequences on GenBank (accessed 13 April 2015), using the search terms “*Trichomonas gallinae*” and “Fe‐ hydrogenase” (*n* = 26). The primers were validated on 10 samples known to be positive for *Trichomonas* infection following successful amplification of the ITS region. All samples were amplified in a 10 µl reaction volume using the same Qiagen Multiplex PCR recipe as above.

### 2.6 PCR amplification of apicomplexan cytochrome *b* region

PCR amplification of the cytochrome *b* region of the mitochondrial genome was used to detect the presence of haemosporidians within the genera *Plasmodium*, *Haemoproteus* and *Leucocytozoon* from DNA extracted from blood. Primers HMRf and H15730 (Table 2) targeted *Haemoproteus* sp. and *Plasmodium* sp. Samples were amplified in a 10 µl reaction volume using the Qiagen Multiplex recipe above, but with 0.4 µM forward and reverse primer. Primers Leunew1F and LDRd (Table 2) were used to target *Leucocytozoon* sp. The PCR recipe is as for HMRf‐H15730, but with 0.2 µM forward and reverse primer.

### 2.7 Sanger sequencing

A subset of positive PCR *T*. *gallinae* products were purified using Wizard SV Gel & PCR Clean‐Up System (Promega) and sequenced in both directions either by Beckman Coulter Genomics, or on an ABI3730 DNA Analyser (Applied Biosystems) in the Molecular Ecology Laboratory at the University of Sheffield. All other positive samples, and 25 of the same samples (to allow validation of HTS methods) were individually tagged and sequenced on an Illumina MiSeq. Nineteen birds were screened for blood parasites using multiple primer pairs as part of a separate study (see Dunn, Stockdale et al., 2017 for full methods), and the positive samples sent for sequencing by Eurofins Genomics. Full details of which samples were sequenced using which method are provided in Appendix S1.

### 2.8 Library preparation for Illumina sequencing

For samples sequenced on the MiSeq platform (Appendix S1), the protocol of Campbell et al. (2015) was adopted with some modifications. The PCR1 mix was a 25 µl reaction with 10 µl Qiagen Multiplex PCR Master Mix (Qiagen), 2.5 µl of each forward and reverse primer (3 µM) tailed with Illumina sequencing primer sites (F: 5′‐TCTACACGTTCAGAGTTCTACAGTCCGACGATC‐3′ and R: 5′–GTGACTGGAGTTCAGACGTGTGCTCTTCCGATCT‐3′) and 1 µl of DNA. The PCR thermal cycling programs were identical to those previously described for the amplified gene regions (Table 1). Amplicons for each sample were normalised according to the intensity of the PCR product on a 1% agarose gel stained with ethidium bromide. PCR2 added sample‐specific indexes: the 10 µl PCR2 mix had 5 µl QIAGEN Multiplex PCR Master Mix (Qiagen), 1 µl of each Illumina Fi5 and Ri7 indexes (1 µM) whose combination was specific to each well (Integrated DNA Technologies) and 4 µl of the pooled amplicons for each sample from PCR1. The thermal cycler conditions were as follows: 15 min at 95°C, then 10 cycles of 10 s at 98°C, 30 s at 65°C and 30 s at 72°C, finishing with 5 min at 72°C. Samples were normalised after quantifying on a FLUOstar OPTIMA (BMG Labtech) using the QuantiFluor dsDNA system (Promega) following the manufacturer's instructions. The Agencourt AMPure XP system (Beckman Coulter) was used for purification according to the manufacturer's instructions. The purified products were eluted in 15 µl of nuclease‐free TE (10 mM Tris‐HCl, 1 mM EDTA, pH 8.0) with 1.5 µl of 10 mM Tris‐HCl/0.05% Tween 20 (pH 8.0) added. The prepared libraries were checked on the Agilent 4200 Tapestation (Agilent Technologies) for the expected peak amplicon size.

Quantification of each pooled product was performed using qPCR. Triplicate dilutions of 1:100, 1:1000 and 1:10,000 of the libraries were produced by serial dilution. The reagent mix and thermal cycling conditions were performed as per the manufacturer's instructions (KAPA library quantification kit; KAPA Biosystems). A StepOnePlus Real‐Time PCR system (Applied Biosystems) was used to run the qPCR. The concentration of each library was calculated using the KAPA data analysis template and normalised to 4 nM. The library was sequenced using 250 paired‐end reads on a MiSeq benchtop sequencer (Illumina). Illumina samples were sequenced over four different Miseq runs, in combination with other samples, as part of a larger project (Thomas, 2017). Each run contained at least 10% duplicates (i.e., 10 duplicates per 96 well plate).

### 2.9 Sequence analysis

Sanger sequences were manually assessed for errors, trimmed and aligned in Bioedit (Hall, 2005). Each sequence was queried using the ncbi‐blast algorithm (Altschul et al., 1997) to determine the closest sequence match. Poor quality sequences (less than 180 bp for ITS region, double peaks throughout the length of the chromatogram or a sequence failing in one direction) were removed from further analysis.

MiSeq sequences were demultiplexed into sample files according to Fi5 and Ri7 indexes by the Illumina miseq control software (v2.5.0.5). trimmomatic v0.36 (Bolger et al., 2014) was used to remove Illumina adapter sequences, low quality bases in the leading or trailing ends and low quality sequences that did not meet the minimum Phred quality score of 20 or the minimum length of 100 bp. Paired end reads were aligned using flash 1.2.11 (Magoč & Salzberg, 2011); sequences that did not meet the minimum length of 250 bp were discarded. Sequences were demultiplexed according to the primer sequences using jmhc and the output file gave sequence variant depths quantified among amplicons (Stuglik et al., 2011). Because we needed to detect differences in strains as small as 1 bp, we used an approach known as the degree of change (DOC) to distinguish between biologically accurate sequences and artefacts, based on the frequency of sequence variants found per sample (Lighten et al., 2014): calculations were performed in a custom Excel macro (Lighten et al., 2014). A variant had to be present in at least 50 copies within an amplicon to be retained within the analysis. This value was chosen to minimize the risk of false positives. All remaining sequences were queried using the ncbi‐blast algorithm (Altschul et al., 1997) to determine the closest sequence match. In the case of the fragmented *Fe*‐*hyd* gene, the four fragments from a sample were overlapped to form the full sequence. Partial *Fe*‐*hyd* sequences were identified where possible by aligning them with full length *Fe*‐*hyd* and sequences downloaded from GenBank for primer design using clustalw (Larkin et al., 2007) in bioedit (Hall, 2005). A neighbour‐joining tree based on this trimmed alignment was constructed in mega6 (Tamura et al., 2013). If a query sequence grouped with a recognized strain (either from GenBank or the full length sequences from this study) and that group had bootstrap support >50%, it was identified as that strain. All identifications were confirmed by performing alignments of the well‐supported groups to check the sequences were identical. Due to low levels of variation in fragments 1 and 2, fragments 3 and 4 were required to distinguish between *Fe*‐*hyd* subtypes, either together, or singularly, with fragments 1 and 2. Samples with multiple copies of fragments 3 and 4 (*n* = 8) were not identified because the combination of fragments could not be determined.

### 2.10 Phylogenetic analyses

To assess phylogenetic relationships between ITS sequences and *Fe*‐*hyd* sequences separately, all available unique ITS (*n* = 19) and *Fe*‐*hyd* (*n* = 39) records were downloaded from GenBank (by using the search terms ‘*Trichomonas gallinae* ITS’ and “*Trichomonas gallinae* Fe hydrogenase or Fe‐hydrogenase or iron hydrogenase”). All downloaded sequences including duplicate strains, along with host species and locations for each are given in Appendices S2 (ITS) and S3 (*Fe*‐*hyd*). Strain nomenclature was adopted from Chi et al. (2013), or the reporting authors for more recently discovered types. Strains were aligned and trimmed to 224 bp (ITS) or 591 bp (*Fe*‐*hyd*). We included *T*. *vaginalis* as an outgroup in both trees; *Tetratrichomonas gallinarum* was also included in the ITS tree, but no *Fe*‐*hyd* sequence was available.

jmodeltest (Darriba et al., 2012) was used to determine the best nucleotide substitution model for ITS (Hasegawa‐Kishino‐Yano plus gamma) and *Fe*‐*hyd* (Hasegawa‐Kishino‐Yano plus gamma) separately, using Bayesian Information Criterion scores. Priors were defined using beauti v1.10.4 (Drummond et al., 2016) including a strict clock and a Yule speciation process (as per Quillfeldt et al., 2018). We constructed Bayesian phylogenetic trees using beast v1.10.4 (Suchard et al., 2018) using Markov Chain Monte Carlo simulations with 25,000,000 generations, sampled every 1000 generations with a 10% burnin. Effective sample sizes >200 and convergence of parameters were confirmed using tracer v1.7.1 (Rambaut et al., 2018) and a maximum clade credibility tree was created in treeannotator v1.10.4.

### 2.11 Statistical analyses

#### 2.11.1 Spatiotemporal strain variation

To test whether the prevalence of each strain differed between years and countries (both specified as categorical variables), we used binomial general linear models (GLMs) in R version 3.3.2 “Sincere Pumpkin Patch” (R Core Team, 2016) with the response variable being the presence or absence of a strain; thus, these analyses only included samples for which we had good quality ITS sequence data (*n* = 128, including previously sequenced samples from the UK in 2011 and 2012 (Lennon et al., 2013; Stockdale et al., 2015), and excluding samples from Burkina Faso because the number of successfully sequenced samples was small (*n* = 4). We initially tested the significance of each term against the null model (containing the response variable and no predictor variables), including the term if *p* > .05; if both Year and Country terms were included, we subsequently tested the removal of each term against the full model. We then carried out post‐hoc pairwise contrasts within the final model to identify where differences lay.

#### 2.11.2 Coinfection

To test whether coinfecting parasite strains (of *Trichomonas* sp. *n* = 5 strains; *Haemoproteus* sp. *n* = 3; and *Leucocytozoon* sp. *n* = 6) occurred together at random, we used the *cooccur* package (Griffith et al., 2016) in R version 3.3.3 “Another Canoe” to analyse data from UK birds. This tests whether the observed frequency of strain cooccurrence is greater or less than expected given the overall prevalence of each strain in the population (Griffith et al., 2016). We also ran the same analysis on the entire data set for which *Trichomonas* sequence data were available (*n* = 128 birds) to quantify the expected levels of *Trichomonas* coinfection given the prevalence of different parasite strains: whilst this data set included sequences identified using Sanger sequencing (where potential coinfections might be missed) no Sanger sequences returned overlapping double peaks in the chromatogram, indicating an absence of coinfection in these samples.

#### 2.11.3 Impacts of coinfection

Rates of coinfection by multiple *T*. *gallinae* strains were low (see Section 3), so we were unable to analyse potential impacts of *T*. *gallinae* coinfection on body condition (e.g., Villanúa et al., 2006). However, we did test whether body condition was influenced by coinfection by *T*. *gallinae* and haemosporidians by fitting a Gaussian GLM using scaled parameters, with weight as the response variable and wing length, time of day, and number of parasite strains (range: 0–5, median: 3) as covariates. We removed individuals showing clinical signs of trichomonosis from this analysis (*n* = 3; Stockdale et al., 2015). We also ran a second GLM as above, replacing the number of parasite strains with a binary coinfected variable, where a coinfected individual was defined as one carrying two or more parasite strains (coinfected *n* = 40; noncoinfected *n* = 5). Post hoc power analyses were conducted using the pwr package (Champely, 2018) in R, using nonscaled parameters to estimate effect sizes, to test the statistical power of both of these analyses.

## 3 RESULTS

### 3.1 *Trichomonas gallinae* sequence identity and method validation

From a total of 185 *T*. *gallinae* positive samples obtained from birds in the UK, France, Senegal and Burkina Faso between 2012/13 and 2015, we obtained good quality ITS sequences from 114 samples (59 were Sanger sequenced and the remaining 55 via Illumina MiSeq), and 29 for *Fe*‐*hyd* (2 Sanger sequenced and 27 via Illumina MiSeq; see Appendix S1 for details of which samples were Sanger sequenced). For Illumina sequencing, genotype repeatability was 100% within the same sequencing run; repeatability between different runs was not tested. Illumina sequence read depths following processing (mean ± SE) per amplicon was 13,662 ± 2671 (ITS), 9852 ± 1530 (Fe‐hyd1), 6634 ± 2825 (Fe‐hyd2), 14,129 ± 2315 (Fe‐hyd3), 11,111 ± 1333 (Fe‐hyd4), 5940 ± 1095 (HMRf – H15730) and 15,373 ± 3972 (Leunew1F – LDRd).

Comparison of Sanger and HTS ITS sequences from the same samples verified that all samples for which both Sanger and HTS returned viable sequence (*n* = 25) reported the presence of the same strain.

**FIGURE 1:**
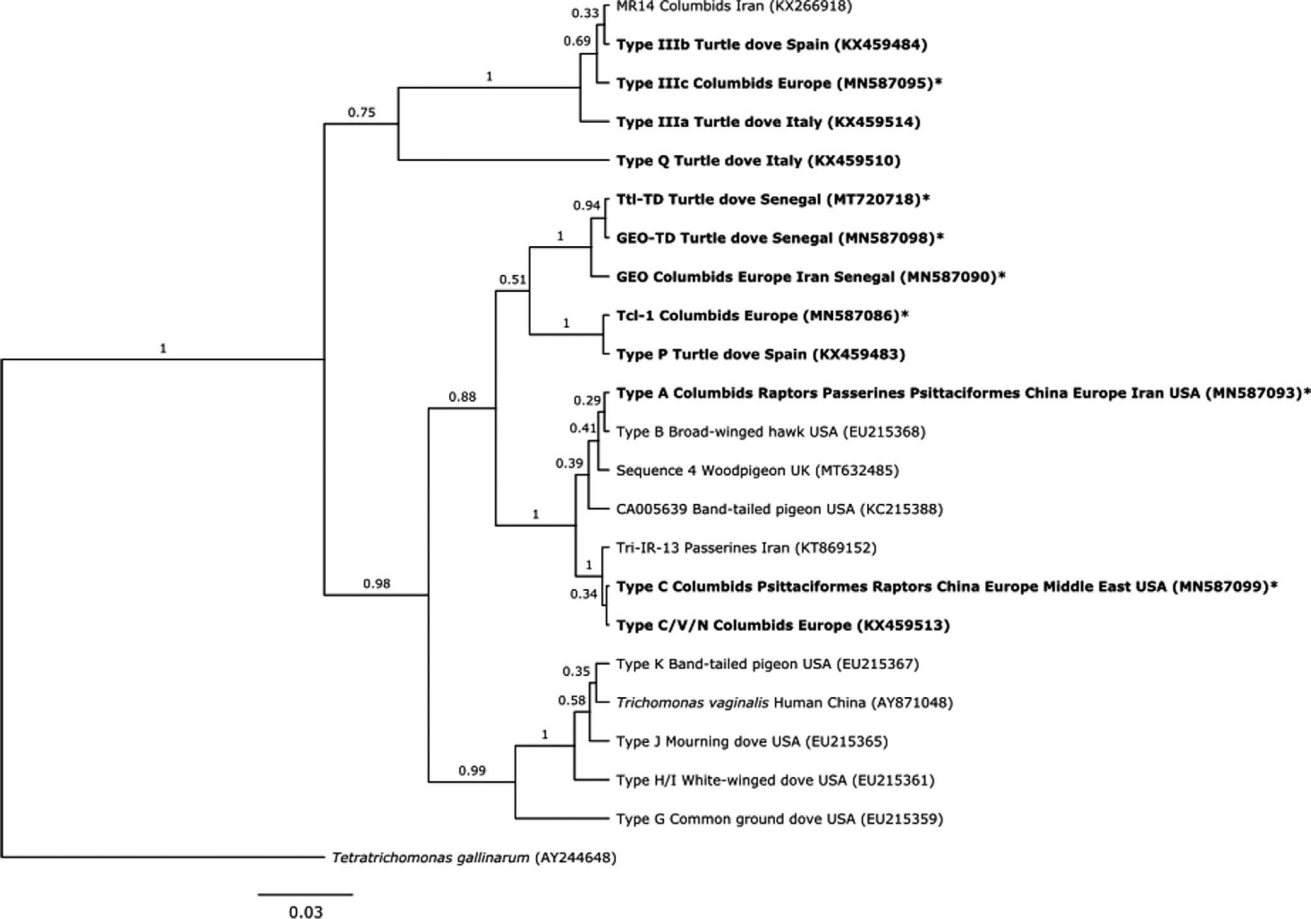
Maximum clade credibility tree of the ITS ribosomal region of all unique *Trichomonas gallinae* sequences from GenBank, using Bayesian inferenced in BEAST. Sequences isolated from turtle doves in our study are marked with a *; all other sequences are labelled with an example GenBank accession number and the full list of sequences analysed is provided in Appendix S2; sequences isolated from turtle doves in our and other studies are emboldened. Also labelled are the hosts and location from which each sequence has been isolated. If a sequence was isolated from more than one species within a family, or more than one country within a continent, the sequence is labelled with that family or continent; if a sequence was isolated from only one species within a family, or only one country within a continent, the sequence is labelled with the species and or country. Branch reliability is provided as a proportion of 1000 bootstrap replicates

Seven distinct ITS sequences were identified. Five were identical to existing strains in GenBank (Figure 1). The sixth sequence was found in one turtle dove from Senegal, representing a new strain with 99% similarity to the “GEO” strain (GenBank accession number (A/N) JQ755287), and is hereafter named GEO‐TD (A/N MN587098). This same new strain was later detected in Laughing Doves *Streptopelia senegalensis* in Senegal (*n* = 2; Thomas, 2017). The seventh sequence was found in four turtle doves from Senegal, representing a new strain with 99.64% similarity to a *T*. *tenax* isolate (A/N KX061780) and is hereafter named Ttl‐TD (A/N MT720718).

**FIGURE 2:**
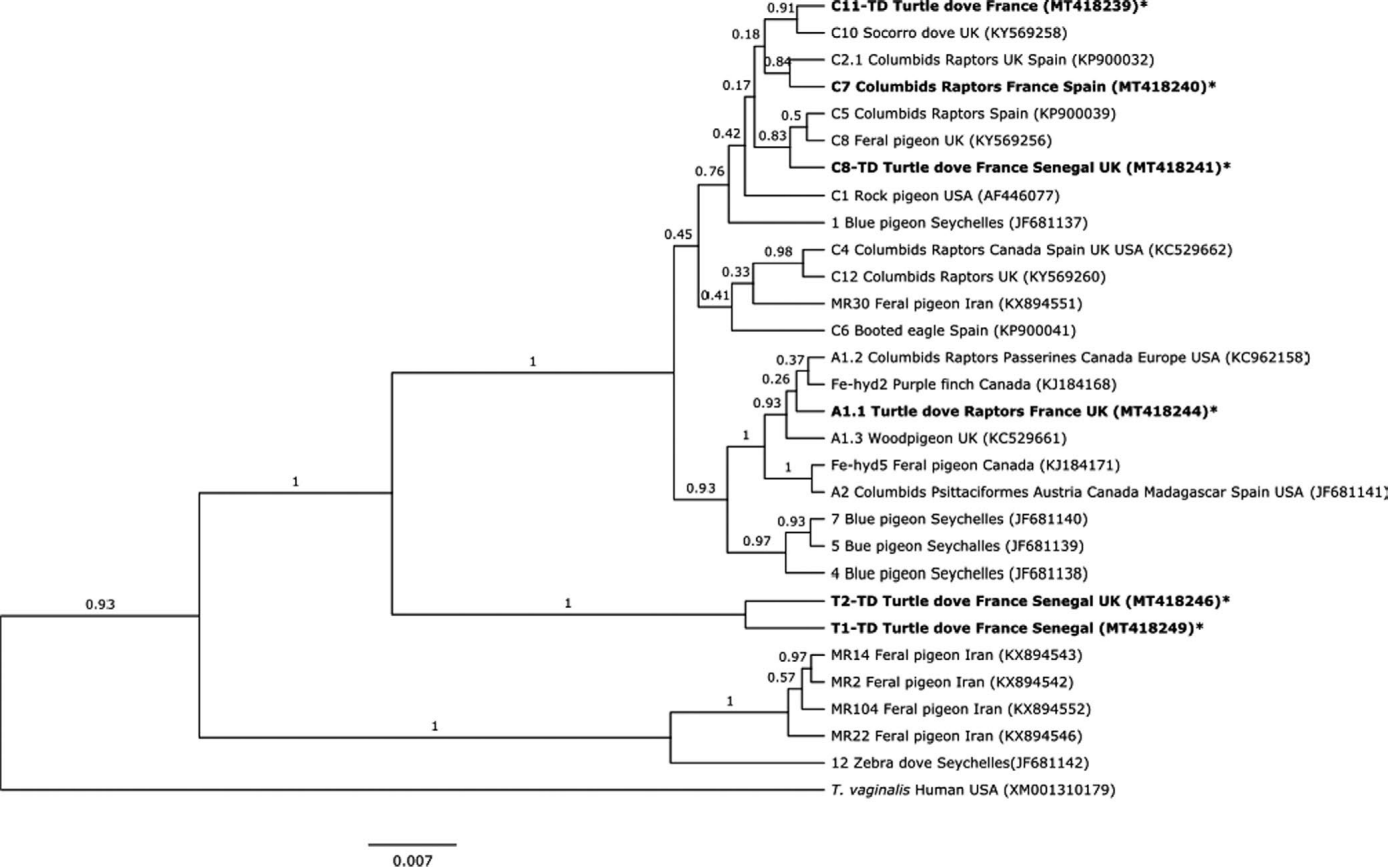
Maximum clade credibility tree of the *Fe*‐*hyd* ribosomal region of all unique *Trichomonas gallinae* sequences from GenBank, using Bayesian inference in BEAST. Sequences isolated from turtle doves in our study are marked with a *; all other sequences are labelled with an example GenBank accession number and the full list of sequences analysed is provided in Appendix S3; sequences isolated from turtle doves in our and other studies are emboldened. Also labelled are the hosts and location from which each sequence has been isolated. If a sequence was isolated from more than one species within a family, or more than one country within a continent, the sequence is labelled with that family or continent; if a sequence was isolated from only one species within a family, or only one country within a continent, the sequence is labelled with the species and or country. Branch reliability is provided as a proportion of 1000 bootstrap replicates

Six *Fe*‐*hyd* variants were identified, two of which were identical to sequences in GenBank (Figure 2). The remaining four represent new subtypes. Two sequences sit within the type C clade: one from 14 birds sampled in 2014 (France, Senegal and UK), hereby labelled C8‐TD (A/N MT418241–43); and the other from one individual in France, 2014, hereby labelled C11‐TD (A/N MT41823). Two new Tcl‐1 subtypes were detected: one from two birds in France and a bird in Senegal 2014, hereby labelled T1‐TD (A/N MT418249–50); and the other from four individuals in France and Senegal, 2014 and UK 2015, hereby labelled T2‐TD (A/N MT418246–48).

### 3.2 Haemosporidia prevalence and method validation

We detected nine haemosporidian lineages, representing *Haemoproteus* sp. (*n* = 3) and *Leucocytozoon* sp. (*n* = 6) (Table 3); no *Plasmodium* infections were detected. All three *Haemoproteus* sp. lineages matched sequences on GenBank with 100% cover and 100% identity (Table 3), including those previously found in nestling turtle doves in the UK (Dunn, Stockdale, et al., 2017); one *Haemoproteus* lineage was amplified using the *Leucocytozoon* primer set, and also matched MalAvi strain STRORI01. The six *Leucocytozoon* sp. lineages matched four strains on the MalAvi database in addition to sequences on GenBank (Table 3). All of these *Leucocytozoon* lineages – including two lineages novel in this system – had been previously reported from nestling turtle doves in the UK (Dunn, Stockdale et al., 2017; Table 3). Twelve samples were infected by multiple *Haemoproteus* (two lineages: *n* = 7) or *Leucocytozoon* (two lineages: *n* = 5; three lineages: *n* = 1) lineages, with 21 samples infected by both *Haemoproteus* and *Leucocytozoon*. Four individuals carried three separate haemoparasite lineages, with one individual carrying four lineages: two *Haemoproteus* and two *Leucocytozoon*. Thirteen samples positive for *Haemoproteus* infection and eight samples positive for *Leucocytozoon* infection were also compared with Sanger sequences obtained as part of a separate project using multiple primer pairs (as per Dunn, Stockdale et al., 2017) to validate the strains detected by HTS (Dunn et al., unpublished data). For those infected with *Haemoproteus*, 12 sequences were detected by both Sanger sequencing and HTS, three sequences were detected by HTS that were not detected by Sanger sequencing and five sequences were detected by Sanger sequencing that were not detected by HTS. For those infected with *Leucocytozoon*, two sequences were detected by both Sanger sequencing and HTS, six sequences were detected by HTS that were not detected by Sanger sequencing and two sequences were detected by Sanger sequencing that were not detected by HTS.

**TABLE 3:** Haemoparasite lineages detected as part of this study, with their closest matches on MalAvi and GenBank databases. All sequences had 100% overlap with their closest match on GenBank. Where MalAvi match and identity = NA, the amplified region does not overlap with the MalAvi barcode region

Lineage (this study)	Parasite genus	MalAvi match	% identity	GenBank match	% identity	*N*	Citation
HB‐TD	*Haemoproteus*	NA	NA	AB741490	100	12	Yoshimura et al. (2014)
				KX832608	100		Dunn, Stockdale et al., 2017
				KX832606	100		Dunn, Stockdale et al., 2017
				KX832604	100		Dunn, Stockdale et al., 2017
				KX832570	100		Dunn, Stockdale et al., 2017
				KX832567	100		Dunn, Stockdale et al., 2017
HC‐TD	*Haemoproteus*	NA	NA	KX832602	100	10	Dunn, Stockdale et al., 2017
HD‐TD	*Haemoproteus*	STRORI01	100	KX832569	100	1	Dunn, Stockdale et al., 2017
				KX832568	100		Dunn, Stockdale et al., 2017
				LC428005	100		K. Tanaka, D. Sumiyama, T. Kanazawa, Y. Sato and K. Murata (unpublished data)
LA‐TD	*Leucocytozoon*	AEMO02	100	KX832556	100	2	Dunn, Stockdale et al., 2017
				KX832555	100		Dunn, Stockdale et al., 2017
				KT779209	100		Y. L. Huang, S. S. Tsai, J. M. Ciou and H. Y. Wu (unpublished data)
				KJ488804	100		Drovetski et al. (2014)
				HF543617	100		Pérez‐Rodríguez et al. (2013)
LB‐TD	*Leucocytozoon*	STRORI02	100	KX832597	100	7	Dunn, Stockdale et al., 2017
				AB741508	100		A. Yoshimura, M. Ko‐ketsu, Y. Watanabe and S. Fukumoto (unpublished data)
LD‐TD	*Leucocytozoon*	AEMO02	99	KX832556	99	1	Dunn, Stockdale et al., 2017
				KX832555	99		Dunn, Stockdale et al., 2017
				KT779209	99		Y. L. Huang, S. S. Tsai, J. M. Ciou and H. Y. Wu (unpublished data)
				KJ488804	99		Drovetski et al. (2014)
				HF543617	99		Pérez‐Rodríguez et al. (2013)
LE‐TD	*Leucocytozoon*	COLIV04	100	KX832576	100	2	Dunn, Stockdale et al., 2017
				AB741506	100		A. Yoshimura, M. Ko‐ketsu, Y. Watanabe and S. Fukumoto (unpublished data)
LG‐TD	*Leucocytozoon*	CIAE02	100	MH644765	100	1	Couto et al. (2019)
				MH644761	100		Couto et al. (2019)
				MH644760	100		Couto et al. (2019)
				MH644759	100		Couto et al. (2019)
				KY448909	100		S. M. Okanga, G. S. Cumming and J. L. Peters (unpublished data)
				KX832575	100		Dunn, Stockdale et al., 2017
				KX832574	100		Dunn, Stockdale et al., 2017
				KU761603	100		A. Yildirim, A. Inci, A. Ciloglu, O. Duzlu, Z. Onder, A. Gursoy Ergen, B. Dik, S. Bensch and G. Valkiunas (unpublished data)
				KJ488908	100		Drovetski et al. (2014)
				KJ577832	100		Seimon et al. (2016)
				KC962152	100		Ciloglu et al. (2016)
				KC962151	100		Ciloglu et al. (2016)
				HF543631	100		Pérez‐Rodríguez et al. (2013)
				JX418201	100		Silva‐Iturriza et al. (2012)
				EF607287	100		Krone et al. (2008)
LJ‐TD	*Leucocytozoon*	STRORI02	99	KX832597	99	1	Dunn, Stockdale et al., 2017
				AB741508	99		A. Yoshimura, M. Ko‐ketsu, Y. Watanabe and S. Fukumoto (unpublished data)

### 3.3 *Trichomonas gallinae* phylogenetic analysis

The ITS phylogenetic tree (Figure 1) revealed four main groups with high bootstrap support. One contained *T*. *vaginalis* and strain types H/I, J, K and G, none of which we found in turtle doves. The second comprised strains GEO, GEO‐TD and Ttl‐TD as a sister taxon to Tcl‐1 (*T*. *canistome*‐like) and type P. The third comprises types A, B, C, C/V/N, Sequence 4 and two uncharacterised strains Tri‐IR‐13 and CA005639. The final clade contains the three type III strains, along with MR14; type Q appears as an outgroup to the four clades, separate from *Tetratrichomonas gallinarum*.

The phylogenetic tree for *Fe*‐*hyd* (Figure 2) had three main clusters with bootstrap greater than 50%. The first clade contained four unclassified strains found in Feral pigeons in Iran, along with a strain found in Zebra doves *Geopelia striata* in the Seychelles. The second clade contained T1‐TD and T2‐TD, both new from this study. The third clade contained types A and C, with strong support for type A as a subclade containing types A1 and A2 along with two Canadian strains, as a sister subclade to three strains found in Blue pigeons *Alectroenas pulcherrimus* in the Seychelles (Figure 2). Support for type C as a separate subclade was weaker and many relationships within this clade remain unresolved: however, novel strain C8‐TD shared a clade with C8 and C5, novel strain C11‐TD was a sister taxon to C10 found in a Socorro dove *Zenaida graysoni*, and C7 shared a clade with C2.1 (Figure 2).

### 3.4 *Trichomonas gallinae* prevalence and spatiotemporal strain variation

Prevalence of *T*. *gallinae* infection in adult turtle doves from all populations and in all years sampled was very high, reaching 100% in most cases (Table 4). Only one case of coinfection between *T*. *gallinae* strains was detected, in an adult bird caught in France during 2014, which was infected with GEO and type III strains. All cases of infection in adult turtle doves during 2013–2015 were subclinical, with no lesions or other clinical signs observed. The apparent lower prevalence of infection in Burkina Faso (89%) is probably an underestimate due to suboptimal storage conditions of samples prior to DNA extraction.

**TABLE 4:** The number of turtle dove samples tested and found positive for *Trichomonas gallinae* infection in each population and year, along with the number of samples successfully sequenced at ITS and *Fe*‐*hyd* regions. Birds from Burkina Faso were caught during the winter of 2012–2013 so the data are combined; UK samples from 2011 to 2012 are not included here as they are previously published elsewhere

Country	Year	*N* sampled	*N* positive	Prevalence (%)	*N* ITS sequences	*N Fe*‐*hyd* sequences
UK	2013	23	22	96	18	2
UK	2014	10	9	90	6	4
UK	2015	4	4	100	3	2
France	2014	78	78	100	40	18
Burkina Faso	2012/13	19	17	89	4	0
Senegal	2014	11	11	100	6	3
Senegal	2015	44	44	100	37	0
Total		189	185	98%	114	29

**FIGURE 3:**
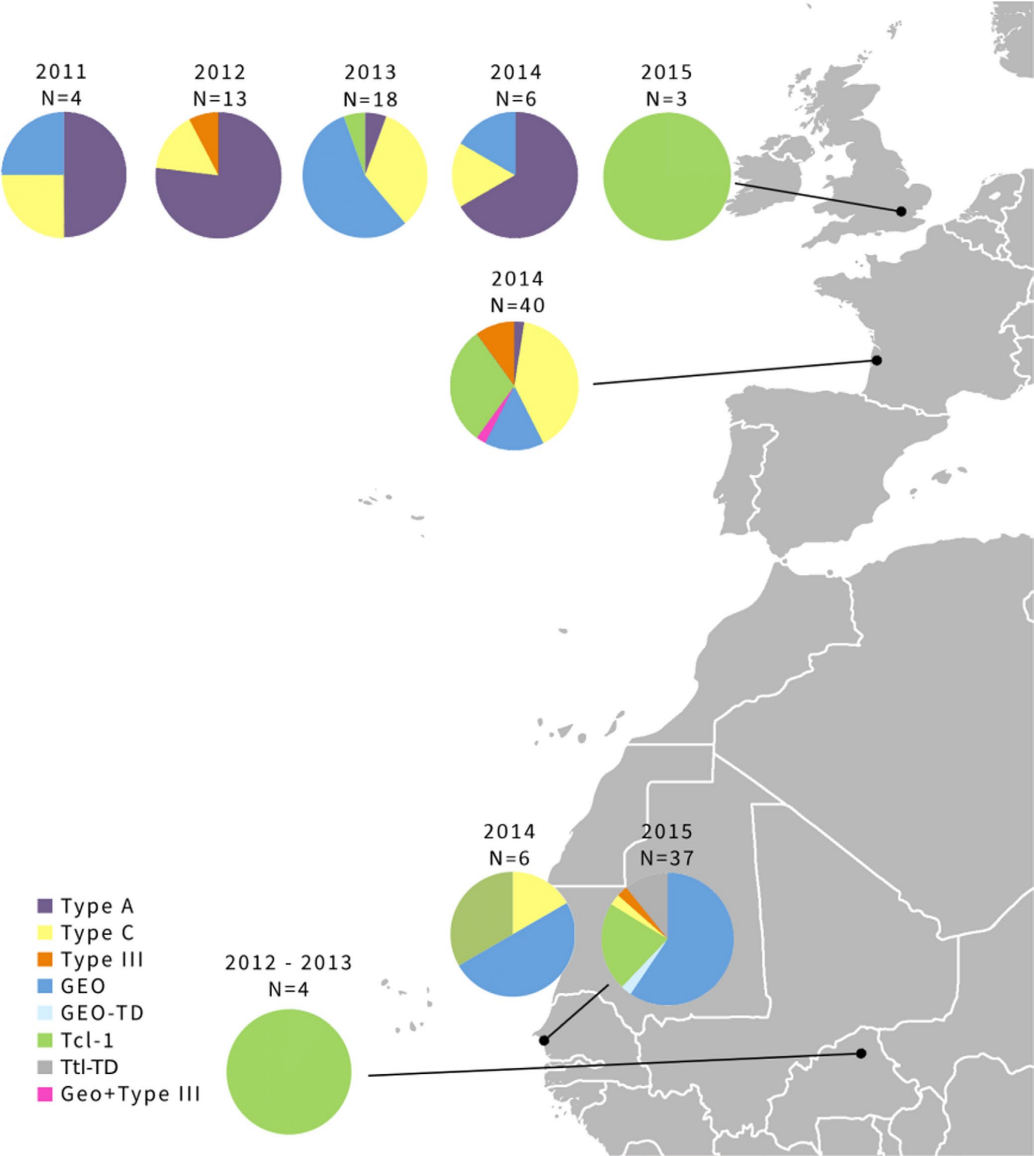
Pie charts reflecting the strain frequency composition of *Trichomonas gallinae* (based on the ITS type) in the sampled populations of turtle doves

**FIGURE 4:**
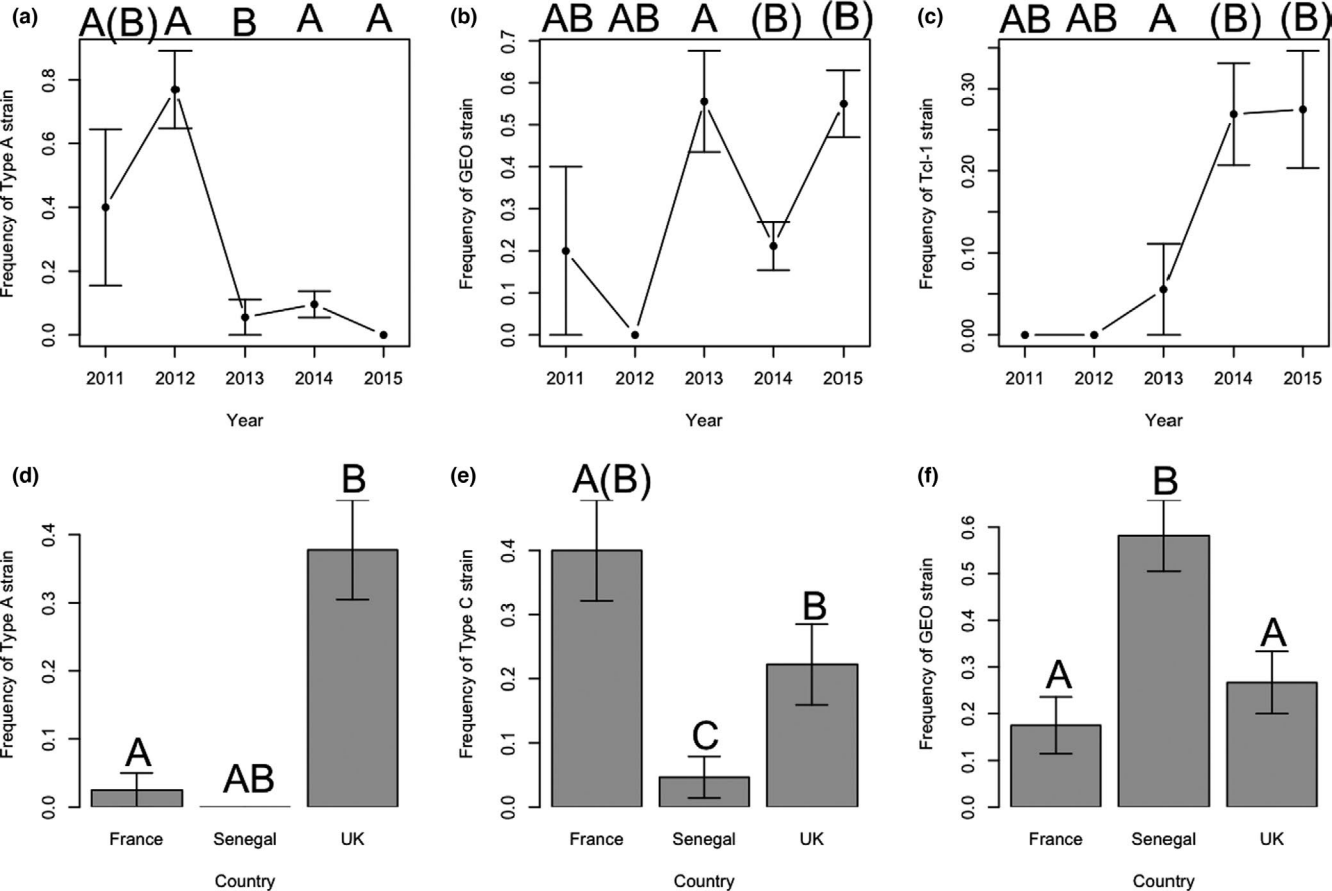
Between year differences in prevalence of *Trichomonas gallinae* strains (a) type A, (b) GEO, and (c) Tcl‐1. Dots show mean values ± SE. Between country differences in prevalence of *T*. *gallinae* strains (d) type A, (e) type C, and (f) GEO. Bars show mean values ± SE. Results from statistical analyses are given in Table 5; letters above bars indicate significant differences at *p* < .05; letters in brackets indicate marginally significant differences at .05 < *p* < .1

*Trichomonas gallinae* ITS haplotype distributions indicated substantial geographical and temporal variation in strain composition in turtle doves (Figure 3; Table 5), with the effects of Year and Country on prevalence differing between strains (Figure 4; Table 5). Type A prevalence was higher in 2011 and 2012 than in 2013–2015, whereas Tcl‐1 prevalence was higher in 2014 and 2015 than previously (Figure 4; Table 5). GEO was absent in 2012, and prevalence was higher in 2013 and 2015 than in 2011 and 2014 (Figure 4; Table 5). Type A, type C and GEO also differed in prevalence between countries, with type A more prevalent in the UK than in France, and absent from Senegal, type C being more prevalent in France than the UK and Senegal, and GEO having higher prevalence in Senegal than either France or the UK (Figure 4; Table 5).

**TABLE 5:** Results of likelihood ratio tests (LRTs) determining whether “Year” and “Country” are significant predictor variables for the variation observed in strain frequency shown in Figure 3 (*n* = 128). Results are from comparisons of the final model with and without the term. Terms in bold were retained in the final model. Dev, deviance. Due to low numbers of positives, analyses were not carried out for GEO_TD (*n* = 1) or Ttl‐TD (*n* = 4). Effect sizes from the raw data are shown in Figures 4 and 5

	Type A			Type C			GEO			Tcl−1			Type III		
	Dev	Df	*p*‐Value	Dev	Df	*p*‐Value	Dev	Df	*p*‐Value	Dev	Df	*p*‐Value	Dev	Df	*p*‐Value
Year	23.53	4	**<.001**	4.62	4	.329	16.20	4	.**002**	13.85	4	.**008**	2.84	4	.584
Country	15.93	2	**<.001**	16.79	2	**<.001**	6.89	2	.**032**	2.25	2	.325	2.94	2	.230

In the UK population, *Fe*‐*hyd* subtypes A1.1 (*n* = 3) and C8‐TD (*n* = 1) were found in 2014, and T2‐TD (*n* = 2) in 2015. Six *Fe*‐*hyd* subtypes were detected in turtle doves in France in 2014, with C8‐TD having the highest prevalence (67%; *n* = 12). Two other type C subtypes were also found in one bird each: C7 and C11‐TD. Type A subtype A1.1 was found in a single bird in France, with two Tcl‐1 subtypes, T1‐TD and T2‐TD, found in two and one birds, respectively. Three different *Fe*‐*hyd* subtypes of Type C and Tcl‐1 were detected in the three turtle doves sampled in Senegal in 2014 (C8‐TD, T1‐TD and T2‐TD).

### 3.5 Coinfections

Coinfection by *T*. *gallinae*, *Haemoproteus* sp. and *Leucocytozoon* sp., was examined in 49 adult turtle doves from the UK between 2011 and 2014 (Table 6a). Only one bird (2%) was negative for all parasites, four birds (8%) were infected only with *T*. *gallinae*, and no birds were infected with only haemosporidia. Most birds were coinfected by all three parasite genera (43%, *n* = 21) or by *T*. *gallinae* and *Haemoproteus* sp. (35%, *n* = 17). Six birds (12%) were coinfected by *T*. *gallinae* and *Leucocytozoon* sp.

**TABLE 6:** (a) Prevalence of coinfection between *Trichomonas gallinae* and haemosporidian parasites between years for UK samples only, and (b) Statistical analysis of coinfection likelihood for strain pairs identified as not randomly associated. The full results for all strain pairs are provided in Appendix S4

(a)					
	2011	2012	2013	2014	Total
No infection	0	0	0	1	1
*Haemoproteus* only	0	0	0	0	0
*Leucocytozoon* only	0	0	0	0	0
*T*. *gallinae* only	0	0	2	2	4
*T*. *gallinae* + Haemoproteus	2	3	9	3	17
*T*. *gallinae* + Leucocytozoon	0	4	2	0	6
*T*. *gallinae* + Haemoproteus + *Leucocytozoon*	4	6	8	3	21
Total	6	13	21	9	49

(b)								
Strain 1	Strain 2	Strain 1 number infected	Strain 2 number infected	Observed number coinfected	Probability of cooccurrence	Expected number coinfected	P Lt	P Gt
Type A	GEO	17	12	0	.085	4.2	.**002**	1.000
Type A	Type C	17	10	0	.071	3.5	.**008**	1.000
Type A	LA‐TD	17	5	4	.035	1.7	.996	.**043**
GEO	Type C	12	10	0	.050	2.4	.**042**	1.000
Type C	LB‐TD	10	11	6	.046	2.2	1.000	.**005**
Type C	LE‐TD	10	4	3	.017	0.8	.999	.**023**
LB‐TD	LE‐TD	11	4	4	.018	0.9	1.000	.**002**
HD‐TD	LD‐TD	1	1	1	.000	0.0	1.000	.**020**

Note

This table shows the number of birds infected with either strain for each pairwise comparison, and the number of birds coinfected with both, along with the probability of cooccurrence based on the occurrence of each strain within the population and expected number of coinfections. P Lt and P Gt represent the probabilities that these species could cooccur less (P Lt) or more often (P Gt) than observed in our data, respectively, by chance. Significant deviations from random (where *p* < .05) are highlighted in bold. GEO, Type A and Type C are *T*. *gallinae* strains; HD‐TD is *Haemoproteus* and LA‐TD, LB‐TD, LD‐TD and LE‐TD are *Leucocytozoon*.

**FIGURE 5:**
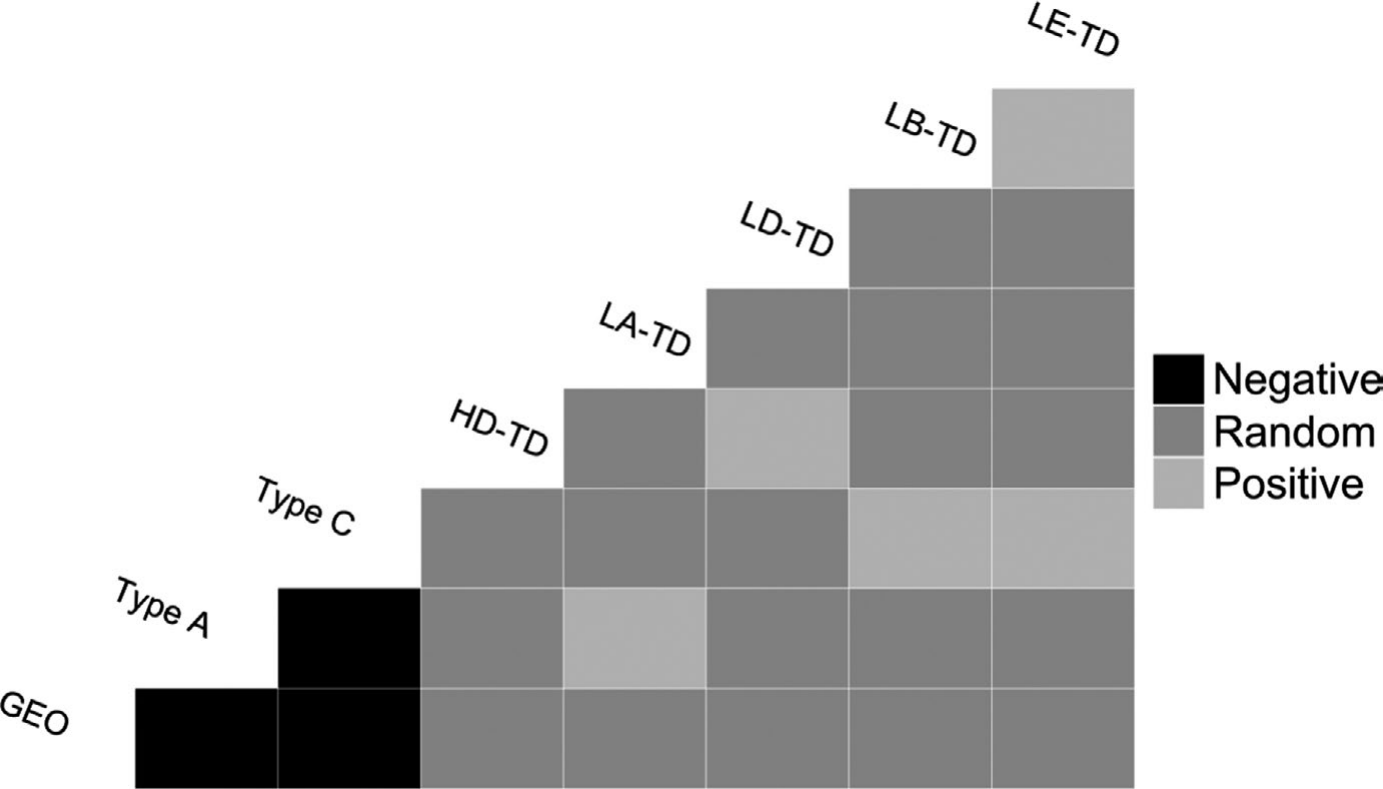
Cooccurrence matrix for parasite strains showing nonrandom associations between *Trichomonas gallinae* (GEO, type A and type C), *Haemoproteus* (HD‐TD) and *Leucocytozoon* (LA‐TD, LB‐TD, LD‐TD and LE‐TD) strains in UK breeding turtle doves

Strain information was gained from a subset of coinfected samples (*T*. *gallinae*, *n* = 41; *Haemoproteus*, *n* = 29; *Leucocytozoon*, *n* = 18) from the UK population only. Both positive and negative associations were found between coinfecting parasite strains (Table 6b; Appendix S4). Negative associations were found between *T*. *gallinae* strains type A, GEO and type C, but positive associations were found between *T*. *gallinae* type A and *Leucocytozoon* LA‐TD, and between *T*. *gallinae* type C and both *Leucocytozoon* LB‐TD and LE‐TD (Figure 5). *Leucocytozoon* LE‐TD was only found in coinfections with LB‐TD, and *Haemoproteus* HD‐TD and *Leucocytozoon* LD‐TD were only found in coinfections with the other. With the exception of type A and LA‐TD, all significant associations were found in multiple years.

Across our entire data set (*n* = 128), only one bird was found to carry more than one strain of *Trichomonas*, with six out of 10 pairwise strain combinations being observed less frequently than expected (Table 7). The observed <1% coinfection prevalence is markedly less than the 38.6% expected given the prevalence of individual strains (Table 7).

**TABLE 7:** Statistical analysis of coinfection likelihood for *Trichomonas* strain pairs for all birds where strain identity was confirmed (*n* = 118). The novel GEO‐TD strain was excluded from analysis because it was only found in a single bird

Strain 1	Strain 2	Strain 1 number infected	Strain 2 number infected	Observed number coinfected	Probability of cooccurrence	Expected number coinfected	P Lt	P Gt
Type A	Type C	20	31	0	.045	5.3	.**001**	1.000
Type A	GEO	20	39	0	.056	6.6	.**001**	1.000
Type A	Tcl‐1	20	22	0	.032	3.7	.**012**	1.000
Type A	Type III	20	7	0	.010	1.2	.262	1.000
Type C	GEO	31	39	0	.087	10.2	.**001**	1.000
Type C	Tcl‐1	31	22	0	.049	5.8	.**001**	1.000
Type C	Type III	31	7	0	.016	1.8	.111	1.000
GEO	Tcl‐1	39	22	0	.062	7.3	.**001**	1.000
GEO	Type III	39	7	1	.020	2.3	.261	.945
Tcl‐1	Type III	22	7	0	.011	1.3	.226	1.000

Note

This table shows the number of birds infected with either strain for each pairwise comparison, and the number of birds coinfected with both, along with the probability of cooccurrence based on the occurrence of each strain within the population and expected number of coinfections. P Lt and P Gt represent the probabilities that these species could cooccur less (P Lt) or more often (P Gt) than observed in our data, respectively, by chance. Significant deviations from random (where *p* < .05) are highlighted in bold.

### 3.6 Impacts of coinfection between *T*. *gallinae* and haemosporidians

We found no evidence for an effect of an increasing number of parasite strains (GLM, *t*_1_ = −0.558, *p* = .580) or the presence of coinfection (GLM, *t*_1_ = −0.114, *p* = .91; full model outputs are provided in Appendix S5) on body condition. Post hoc power analyses suggested our analyses had sufficient power to detect small (0.1; power = 0.526), medium (0.3; power = 0.939) and large (0.5; power = 0.995) effect sizes.

## 4 DISCUSSION

Coinfecting parasites can reduce host survival (Davidar & Morton, 2006) and reproductive success (Bunbury, 2006), as well as influencing an individual's chance of acquiring new infections (Telfer et al., 2010). Despite this, coinfections in wild populations tend to be overlooked, often due to methodological difficulties in their identification. Here, we develop and use new methods to identify coinfections within and between multiple parasite taxa. Contrary to our predictions, we found both spatial and temporal variation between populations across multiple years. We also found a surprisingly low prevalence of coinfection by multiple *T*. *gallinae* strains in turtle doves, despite a high overall prevalence and diverse strain composition within populations. In contrast, we found a high prevalence of coinfection by multiple haemoparasites in UK breeding turtle doves, with suggestions of antagonistic, facultative and neutral associations between different parasite strains.

### 4.1 Parasite prevalence and *T*. *gallinae* spatiotemporal strain variation

Prevalence of *T*. *gallinae* and haemosporidian infection was very high in all turtle dove populations sampled, with multiple *T*. *gallinae* strains circulating in all populations. Our results are concordant with other recent studies of columbids, which found an 86% prevalence of *T*. *gallinae* infection in both turtle doves and Eurasian collared doves *Streptopelia decaocto* in the UK (Lennon et al., 2013). A high prevalence of haemosporidian infection was also found in nestling turtle doves from the populations in which we sampled adults (Dunn, Stockdale, et al., 2017). A 92% prevalence of *T*. *gallinae* infection has also been reported from turtle doves in Spain, Italy and Germany (Marx et al., 2017). For *T*. *gallinae*, all HTS sequences matched with Sanger sequences where samples were sequenced using both methods, and HTS repeatability was 100% which suggests that HTS, when combined with the pipeline that we used, is a reliable method to detect and identify *T*. *gallinae* infections. Haemosporidian HTS sequences were less reliable, with both Sanger and HTS detecting strains not detected by the other. This is probably due to differences between the sequences of the primer sets we used for HTS and Sanger sequencing. For HTS we used two existing haemosporidian primer sets (Fallon et al., 2003; Martínez et al., 2009; Merino et al., 2008; Quillfeldt et al., 2014) that produced suitable length amplicons, rather than designing new bespoke primers to detect all strains present. That strains were detected using HTS that were not detected using Sanger sequencing (using multiple primer sets) suggests that HTS, with a refined selection of bespoke universal primers, may be a very useful tool for detecting cryptic coinfections where only the dominant strain is amplified using Sanger sequencing (Bernotienė et al., 2016).

We found differences in *T*. *gallinae* strain composition between turtle dove populations on breeding and wintering grounds, with type A more prevalent in the UK than France, and not detected in Senegal, type C more prevalent in France than the UK and Senegal, and the GEO strain more common in Senegal. Based on telemetry data, the individuals sampled on wintering grounds could belong to either the French or the UK breeding populations (Eraud et al., 2013; H. Lormée and C. Eraud, unpublished data; S. Requena, H. Lormee, C. J. Orsman, C. Eraud, G. Buchanan, A. Beresford, M. Riviére, J. A. Vickery and J. W. Mallord, unpublished data), so do not necessarily represent distinct populations. It is also possible that some birds sampled in Senegal belong to the north African subspecies *S*. *turtur arenicola*, which shares wintering grounds with the European breeding nominate *S*. *turtur turtur*: subsequent analysis of morphometric data suggests around 16% of turtle doves sampled in Senegal are European breeding *S*. *t*. *turtur*, 56% are *S*. *t*. *arenicola*, and 27% could not be assigned to subspecies based on morphological measurements (Cramp, 1980). We know very little about *T*. *gallinae* strain turnover within individuals, so it may be that birds gain and lose strains relatively rapidly from their environment depending on the strains they are exposed to via food and/or water sources, and thus the strain composition we detect may reflect the recent environmental transmission of strains, rather than the exposure history of individuals.

We identified seven *T*. *gallinae* ITS strains, five of which have been reported previously and two of which (GEO‐TD and Ttl‐TD) are novel strains. We also detected six *Fe*‐*hyd* subtypes from three ITS strains, of which four are novel. This strain diversity is comparable to other studies of *T*. *gallinae* that have included turtle doves, with Marx et al. (2017) and Martínez‐Herrero et al. (2014) detecting types C, V, II, III plus two novel strains in Germany and Spain. Interestingly, neither of these studies detected the type A strain, believed to be a major driver of greenfinch *Chloris chloris* population decline in the UK (Lawson et al., 2018), in turtle doves, although Marx et al. (2017) did detect this strain in other columbid species. Infection with this strain may be short‐lived in turtle doves, as they either clear it or die (Stockdale et al., 2015), and therefore it does not seem to have reached populations further from the UK, where it is thought to have emerged (Lawson et al., 2012).

This study is the first to assess temporal variation in *T*. *gallinae* strain composition, covering a five‐year span in the UK. We found significant variation in strain composition between years, suggesting a relatively rapid strain turnover in the population. In particular, type A occurred in much lower frequencies in 2013–2015 than previously. In 2012 there was unusually high summer rainfall (Met Office, 2016) and *T*. *gallinae*‐associated mortality was detected in the UK turtle dove population (Stockdale et al., 2015). Turtle doves in the UK are known to use supplementary food sources, such as grain spillages and garden bird feeders (Browne & Aebischer, 2003; Dunn et al., 2018): the wet conditions in summer 2012 are likely to have reduced natural food availability for a range of farmland bird species (Walker et al., 2018) and thus to have increased reliance on supplementary seed sources, as well as increasing the survival of *T*. *gallinae* on these seed sources (McBurney et al., 2017). The wet weather may also have depressed immune function across species, reducing resistance to infection (Lifjeld et al., 2002). As a result of this, cross‐species *T*. *gallinae* transmission is likely to have increased. Associated work found the type A strain to be particularly associated with supplementary food sources (Thomas, 2017), suggesting this strain may be more effective at being transmitted via the environment than other strains. Given the potential population impacts of type A infection due to its high virulence (Stockdale et al., 2015), management of supplementary food resources to minimise transmission risks, especially during wet conditions, is imperative. The key element is likely to be reducing the density of birds feeding at any single source: for example rotating the locations of multiple food sources, or scattering seed to prevent high densities of birds congregating in one area, may be key to preventing parasite build‐up in one place and thus reducing transmission.

### 4.2 Coinfections

The likelihood of coinfection by multiple strains of *T*. *gallinae* depends on the circulation of multiple strains in a population, and parasite transmission routes. With only one case of *T*. *gallinae* strain coinfection, our results suggest that coinfection is rarer than previously suggested by Grabensteiner et al. (2010), who reported a rate of 12% in pigeons (*n* = 17). Indeed, we observed coinfection by multiple *T*. *gallinae* infections in <1% of birds, occurring at significantly lower frequencies than the expected coinfection rate of 38.6% given the strain diversity we observed, and restricting our ability to directly test for any negative impacts of coinfection on body condition. Here, we demonstrate that multiple strains circulate in all the turtle dove populations sampled, and thus some mechanism appears to be acting to reduce multiple strains from being present within the same bird. *T*. *gallinae* is transmitted both horizontally at shared food and water sources (McBurney et al., 2017; Purple & Gerhold, 2015), and vertically from parent to offspring, through regurgitated crop milk (Stabler, 1954). In terms of vertical transmission, if both parents carry the same single *T*. *gallinae* strain then nestlings will have single strain infections. We had only two cases where we sampled both individuals within a breeding pair, but one of these pairs carried two strains of *T*. *gallinae* supporting the idea that nestlings may be exposed to multiple strains whilst in the nest. Horizontal transmission of *T*. *gallinae* via shared environmental resources is a more recently proposed transmission route, used to explain the spillover of *T*. *gallinae* to novel passerine hosts (Anderson et al., 2009; Lawson et al., 2012; Stockdale et al., 2015) and we show elsewhere that multiple strains can be present within the same environmental resource using the same methodology (Thomas, 2017). Environmental transmission has the potential to increase the exposure of a host to multiple strains if they utilize shared resources (Jones et al., 2013). If exposure to multiple strains is likely, as suggested by the high strain diversity in all our populations, there are four options to explain our observation of low coinfection rates. First, the low prevalence of coinfection could be explained by an antagonistic interaction between *T*. *gallinae* strains. If occurring, competitive exclusion must act relatively rapidly in our system, and would also explain the difference between populations where probably overlapping strains occur if strain composition reflected exposure within the recent environment, rather than each individual being infected with a single strain and lifelong infection; little is known about the rate of strain turnover within individual birds. Second, there may be immune modulated (apparent) competition between similar parasite strains. Since multiple strains of the same parasite are likely to be competing for the same resources, one strain may reduce survival of intrahost competitors, which would lead to an increase in the fitness of that strain (Fenton et al., 2010). Third, it is possible that competition occurs within the InPouch kits we use to culture *T*. *gallinae* strains, rather than in the bird. However, elsewhere, we found high levels of coinfection in environmental resources using the same technique (Thomas, 2017), and HTS should still enable us to detect DNA from the outcompeted strain, albeit at lower levels. Indeed, a subsequent study has found higher levels of coinfection using the same techniques (R. E. Young, J. C. Dunn, I. P. Vaughan, J. W. Mallord, L. E. Drake, C. J. Orsman, M. Ka, M. B. Diallo, M. Sarr, H. Lormée, C. Eraud, O. Kiss, A. Marchbank and W. O. C. Symondson, unpublished data), suggesting this is unlikely to be the reason behind the low levels of coinfection we see here. Finally – and with potentially significant impacts for turtle dove populations – coinfection by multiple *T*. *gallinae* strains may increase host mortality, as found with respiratory infections (Sid et al., 2015), and with multiple *Plasmodium* strains (Palinauskas et al., 2011). This would result in coinfected individuals being removed from the population, having significant implications for the conservation of turtle dove populations. However, this would ideally need to be tested either through longitudinal studies where the same individuals could be captured on multiple occasions, or experimentally using captive doves.

Conversely, the relatively high prevalence of haemosporidian coinfection reflects the high strain diversity and prevalence of haemosporidian diversity in the UK population. A recent finding of 30% prevalence in 7‐day old nestlings (Dunn, Stockdale, et al., 2017) suggests that multiple strains infect nestling turtle doves on breeding grounds, but as a migratory bird, they may be exposed to a greater diversity of parasites associated with the various habitats utilised over their annual cycle (Figuerola & Green, 2000). Coinfection by multiple species of haemosporidians also depends on exposure to vectors – which may also be coinfected, resulting in cotransmission – which is facilitated by environmental conditions (Cosgrove et al., 2008; van Rooyen et al., 2013), so the prevalence of coinfection with haemosporidians may vary with annual climatic variation that influences vector abundance or activity. Haemosporidian infections are frequently lifelong following initial infection, with relapses occurring on an annual basis at the onset of breeding (Valkiūnas, 2005), increasing the likelihood of coinfections persisting within an individual. Although *T*. *gallinae* coinfection is rare in our turtle dove populations, the prevalence of coinfection between *T*. *gallinae* and either *Haemoproteus* sp. or *Leucocytozoon* sp. parasites in the UK population is high. Persistence of coinfection between *T*. *gallinae* and haemosporidians could potentially be explained by an absence of interaction if they exploit different host resources. Alternatively, there could be synergistic interactions between parasites, such as parasite‐induced immune suppression (Clark et al., 2016; Cox, 2001; Fenton et al., 2010; Graham, 2008) where infection with one parasite predisposes the host to infection by another. We found no evidence of any negative impacts of coinfection by *T*. *gallinae* and blood parasite strains on turtle dove body condition, despite a high statistical power to detect such an effect. Future work could examine more sensitive metrics such as immune response to comprehensively test whether and how coinfection by multiple parasites influences the host.

## 5 CONCLUSION

Monitoring parasite communities, even those that cause no apparent clinical signs, is imperative if we are to predict and mitigate against potential emerging infectious diseases. Here, we provide evidence that HTS can be an efficient method for detecting multiple strains of coinfecting parasites, when appropriate universal primers are selected. We found a high prevalence of coinfection between multiple haemosporidian strains, and between haemosporidians and *T*. *gallinae*, although no apparent negative associations between coinfection and host body condition. However, we did find an unexpectedly low prevalence of coinfection with multiple *T*. *gallinae* strains despite overall high prevalence and high strain diversity across multiple populations, suggesting that either within‐host competition between strains, or high host mortality may result from *T*. *gallinae* coinfections. The latter scenario would have significant implications for turtle dove conservation management, and warrants further investigation. For turtle doves, we suggest that careful management of supplementary food is critical to reduce the risk of transmission and thus infection by multiple *T*. *gallinae* strains.

## AUTHOR CONTRIBUTIONS

Rebecca C. Thomas, Jenny C. Dunn, Antony J. Morris and Simon J. Goodman designed the study, Rebecca C. Thomas, Jenny C. Dunn, Chris Orsman, John Mallord, Cyril Eraud, and Lormée Hervé collected field data, Rebecca C. Thomas, Deborah A. Dawson, Helen Hipperson and Gavin J. Horsburgh contributed to laboratory analysis. Rebecca C. Thomas and Jenny C. Dunn carried out phylogenetic and statistical analyses, and all authors contributed towards writing and editing the manuscript.

## BENEFIT‐SHARING STATEMENT

A research collaboration was developed with scientists from France, with all collaborators included as coauthors or in the acknowledgements, depending on their level of input. The results of the research have been shared with the broader scientific community through appropriate public databases and data repositories (see above). The research addresses a primary concern, in this case the conservation of the turtle dove. Sample collection in the UK was carried out under licence from the Home Office, with bird ringing carried out under licence from the British Trust for Ornithology. Sample collection in western France was carried out thanks to study sites piloted by the French National Game and Wildlife Agency (ONCFS). Research in Burkina Faso was carried out by the RSPB with the permission of the Director of Wildlife and Hunting, and research in Senegal was carried out by the RSPB under a permit granted by Direction des Eaux, Forêts, Chasses et de la Conservation des Sols. Samples were imported to the UK under Defra Import Licences PATH/201/2012/1, PATH/201/2012/2 and PATH/15/0482.

## CONFLICT OF INTEREST

The authors have no conflict of interest.

### OPEN RESEARCH BADGES

This article has earned an Open Data Badge for making publicly available the digitally–shareable data necessary to reproduce the reported results. The data is available at https://doi.org/10.5061/dryad.xwdbrv1fq.

## Supporting information

Supplementary Material

## Data Availability

*Trichomonas gallinae* sequence data are available on GenBank under Accession Numbers MN587086–MN587101, MT418239–MT418250 and MT720718. Data from NGS sequencing runs and metadata are available from the Sequence Read Archive (SRA) under Bioproject accession number PRJNA578480; file accession numbers SRR16955911–967 (*Trichomonas gallinae*) and SRR17676090–131 (blood parasites). Data sets and R code used in analyses are available through Data Dryad at https://doi.org/10.5061/dryad.xwdbrv1fq.
