# Supplementary material for: Assessing rates of parasite coinfection and spatiotemporal strain variation via metabarcoding: Insights for the conservation of European turtle doves Streptopelia turtur
Source: Mol Ecol. 2022 Apr 2;31(9):2730–51. doi: 10.1111/mec.16421 (PMC9325524; doi:10.1111/mec.16421)
Supplement: Supplementary file 1 — Supplementary Material [file MEC-31-2730-s001.pdf]

MOLECULAR ECOLOGY

Supplemental Information for:

Assessing rates of parasite coinfection and spatiotemporal strain variation via metabarcoding: insights for the conservation of European Turtle Doves *Streptopelia turtur*.

Rebecca C. Thomas, Jenny C. Dunn, Deborah A. Dawson, Helen Hipperson, Gavin J. Horsburgh, Antony J. Morris, Chris Orsman, John Mallord, Philip V. Grice, Keith C. Hamer, Cyril Eraud, Hervé Lormée, Simon J. Goodman

Table of Contents:

**Appendix S1**	Page 2
**Appendix S2**	Page 4
**Appendix S3**	Page 17
**Appendix S4**	Page 24
**Appendix S5**	Page 31
**References**	Page 31

MOLECULAR ECOLOGY

Appendix S1. Details of the primers used to amplify samples, type of DNA extraction method and sequencing approach according to year sample and geographic region sample collected. DNeasy = DNeasy blood and tissue kit (Qiagen, Hilden, Germany), A.A method = ammonium acetate method (Nicholls *et al.* 2000).

**Primer set**	**Samples**	**DNA Extraction method**	**Sequencing method**
TFR1-TFR2	UK 2013	DNeasy	Sanger sequencing (Beckman Coulter Genomics)
TFR1-TFR2	UK 2014	A.A method	Illumina MiSeq*
TFR1-TFR2	UK 2015	A.A method	Illumina MiSeq
TFR1-TFR2	France 2014	A.A method	Illumina MiSeq*
TFR1-TFR2	Burkina Faso 2012/13	A.A method	Sanger sequencing
TFR1-TFR2	Senegal 2014	A.A method	Illumina MiSeq*
TFR1-TFR2	Senegal 2015	A.A method	Sanger sequencing^
TrichhydFor-TrichhydREV	UK 2013	DNeasy	Sanger sequencing (Beckman Coulter Genomics)
FeH1 - 4FOR-REV	UK 2014	A.A method	Illumina MiSeq
FeH1 - 4FOR-REV	UK 2015	A.A method	Illumina MiSeq

MOLECULAR ECOLOGY

FeH1 - 4FOR-REV	France 2014	A.A method	Illumina MiSeq
FeH1 - 4FOR-REV	Senegal 2014	A.A method	Illumina MiSeq
HMRf -15730	UK 2011	DNeasy	Illumina MiSeq**
HMRf -15730	UK 2012	DNeasy	Illumina MiSeq**
HMRf -15730	UK 2013	DNeasy	Illumina MiSeq
HMRf -15730	UK 2014	A.A method	Illumina MiSeq
Leunew1F-LDRd	UK 2011	DNeasy	Illumina MiSeq**
Leunew1F-LDRd	UK 2012	DNeasy	Illumina MiSeq**
Leunew1F-LDRd	UK 2013	A.A method	Illumina MiSeq
Leunew1F-LDRd	UK 2014	A.A method	Illumina MiSeq

* A subset of these samples were also sequenced using Sanger sequencing to allow validation of methods

^ A subset of these samples were sequenced using the Illumina MiSeq to allow validation of methods

** These samples were Sanger sequenced as part of a separate study using multiple primer pairs (Dunn et al., 2017; unpubl. data), which allowed validation of methods

MOLECULAR ECOLOGY

Appendix S2. Available ITS sequences downloaded from GenBank (accessed 15/05/2019) and consolidated into strains for construction of Figure 1.

**Accession number**	**Host**	**Location**	**Strain ID**	**Reference**
KC215388*	*Patagioenas fasciata monilis*	USA	CA005639	Girard *et al.* 2014
MN587090*	*Streptopelia turtur*	UK	GEO	This study
MN587091	*Streptopelia turtur*	France	GEO	This study
MN587092	*Streptopelia turtur*	Senegal	GEO	This study
JQ755287	not provided	not provided	GEO	Peters & Raidal unpubl.
KX459478	*Streptopelia turtur*	Spain	GEO	Marx *et al.* 2017
KX459459	*Columba palumbus*	Germany	GEO	Marx *et al.* 2017
KX459511	*Streptopelia turtur*	Italy	GEO	Marx *et al.* 2017
KX459464	*Columba palumbus*	Germany	GEO	Marx *et al.* 2017
KX459461	*Columba palumbus*	Germany	GEO	Marx *et al.* 2017
KX459466	*Columba palumbus*	Germany	GEO	Marx *et al.* 2017

KX459463	*Columba palumbus*	Germany	GEO	Marx *et al.* 2017
KX459457	*Columba palumbus*	Germany	GEO	Marx *et al.* 2017
KX459467	*Columba palumbus*	Germany	GEO	Marx *et al.* 2017
KX459462	*Columba palumbus*	Germany	GEO	Marx *et al.* 2017
KX459465	*Columba palumbus*	Germany	GEO	Marx *et al.* 2017
KX459501	*Streptopelia turtur*	Italy	GEO	Marx *et al.* 2017
KX459479	*Streptopelia turtur*	Spain	GEO	Marx *et al.* 2017
KX459486	*Streptopelia turtur*	Spain	GEO	Marx *et al.* 2017
KX459449	*Columba oenas*	Germany	GEO	Marx *et al.* 2017
KX459456	*Columba palumbus*	Germany	GEO	Marx *et al.* 2017
KX266927	unspecified Pigeon sp.	Iran	GEO	Rajabloo *et al*. unpubl.
KX459453	*Columba oenas*	Germany	GEO	Marx *et al.* 2017
KX459452	*Columba oenas*	Germany	GEO	Marx *et al.* 2017
KX266923	unspecified Pigeon sp.	Iran	GEO	Rajabloo *et al*. unpubl.

KX266922	unspecified Pigeon sp.	Iran	GEO	Rajabloo *et al*. unpubl.
KX266921	unspecified Pigeon sp.	Iran	GEO	Rajabloo *et al*. unpubl.
KX266920	unspecified Pigeon sp.	Iran	GEO	Rajabloo *et al*. unpubl.
KX266919	unspecified Pigeon sp.	Iran	GEO	Rajabloo *et al*. unpubl.
KX266917	unspecified Pigeon sp.	Iran	GEO	Rajabloo *et al*. unpubl.
KX266925	unspecified Pigeon sp.	Iran	GEO	Rajabloo *et al*. unpubl.
KX459504	*Streptopelia turtur*	Italy	GEO	Marx *et al.* 2017
KX459468	*Columba palumbus*	Germany	GEO	Marx *et al.* 2017
MN587098*	*Streptopelia turtur*	Senegal	GEO-TD	This study
KX266918*	unspecified Pigeon sp.	Iran	MR14	Rajabloo *et al*. unpubl.
MN587086*	*Streptopelia turtur*	UK	Tcl-1	This study
MN587087	*Streptopelia turtur*	France	Tcl-1	This study
MN587088	*Streptopelia turtur*	Senegal	Tcl-1	This study
MN587089	*Streptopelia turtur*	Burkina Faso	Tcl-1	This study

KF993705	*Streptopelia turtur*	Spain	Tcl-1	Martínez-Herrero *et al.* 2014
KX459492	*Streptopelia turtur*	Spain	Tcl-1	Marx *et al.* 2017
KX459488	*Streptopelia turtur*	Spain	Tcl-1	Marx *et al.* 2017
KX459481	*Streptopelia turtur*	Spain	Tcl-1	Marx *et al.* 2017
KX459508	*Streptopelia turtur*	Italy	Tcl-1	Marx *et al.* 2017
KX459496	*Streptopelia turtur*	Spain	Tcl-1	Marx *et al.* 2017
KX459512	*Streptopelia turtur*	Italy	Tcl-1	Marx *et al.* 2017
KX459491	*Streptopelia turtur*	Spain	Tcl-1	Marx *et al.* 2017
KX459487	*Streptopelia turtur*	Spain	Tcl-1	Marx *et al.* 2017
KX459502	*Streptopelia turtur*	Italy	Tcl-1	Marx *et al.* 2017
KX459509	*Streptopelia turtur*	Italy	Tcl-1	Marx *et al.* 2017
KX459482	*Streptopelia turtur*	Spain	Tcl-1	Marx *et al.* 2017
KX459451	*Columba oenas*	Germany	Tcl-1	Marx *et al.* 2017
KX459480	*Streptopelia turtur*	Spain	Tcl-1	Marx *et al.* 2017

KX844985	*Streptopelia turtur*	Malta	Tcl-1	Marx *et al.* 2017
KX844987	*Streptopelia turtur*	Malta	Tcl-1	Marx *et al.* 2017
KX459454	*Columba oenas*	Germany	Tcl-1	Marx *et al.* 2017
KX844984	*Streptopelia turtur*	Malta	Tcl-1	Marx *et al.* 2017
KT869152*	*Serinus canaria*	Iran	Tri-IR-13	Ghorbani *et al*. unpubl.
MT720718*	*Streptopelia turtur*	Senegal	Ttl-TD	This study
KX459514*	*Streptopelia turtur*	Italy	Type IIIa±	Marx *et al.* 2017
KX459484*	*Streptopelia turtur*	Spain	Type IIIb±	Marx *et al.* 2017
MN587095*	*Streptopelia turtur*	UK	Type IIIc±	This study
MN587096	*Streptopelia turtur*	France	Type IIIc±	This study
MN587097	*Streptopelia turtur*	Senegal	Type IIIc±	This study
FN433473	*Columba livia*	Austria	Type IIIc±	Grabensteiner *et al.* 2010
KX459495	*Streptopelia turtur*	Spain	Type IIIc±	Marx *et al.* 2017
KX459507	*Streptopelia turtur*	Italy	Type IIIc±	Marx *et al.* 2017

MOLECULAR ECOLOGY

KX459503	*Streptopelia turtur*	Italy	Type IIIc±	Marx *et al.* 2017
KX844989	*Streptopelia turtur*	Malta	Type IIIc±	Marx *et al.* 2017
KX844986	*Streptopelia turtur*	Malta	Type IIIc±	Marx *et al.* 2017
KX459450	*Columba oenas*	Germany	Type IIIc±	Marx *et al.* 2017
KX459448	*Columba oenas*	Germany	Type IIIc±	Marx *et al.* 2017
KC529665	*Caloenas nicobarica*	UK	Type IIIc±	Chi *et al.* 2013
KX459500	*Streptopelia turtur*	Spain	Type IIIc±	Marx *et al.* 2017
KX459506	*Streptopelia turtur*	Italy	Type IIIc±	Marx *et al.* 2017
KX459494	*Streptopelia turtur*	Spain	Type IIIc±	Marx *et al.* 2017
MN587093*	*Streptopelia turtur*	UK	Type A	This study
MN587094	*Streptopelia turtur*	France	Type A	This study
GQ150752	*Carduelis chloris*	UK	Type A	Robinson *et al.* 2010
KX459445	*Columba oenas*	Germany	Type A	Marx *et al.* 2017
KT003194	*Tyto alba*	USA	Type A	Cleveland *et al.* unpubl.

KX459444	*Columba oenas*	Germany	Type A	Marx *et al.* 2017
KX459440	*Columba oenas*	Germany	Type A	Marx *et al.* 2017
KT869157	*Acridotheres tristis*	Iran	Type A	Ghorbani *et al*. unpubl.
KT869155	*Melopsittacus undulatus*	Iran	Type A	Ghorbani *et al*. unpubl.
KJ776742	*Otus scops*	Spain	Type A	Martínez-Herrero *et al.* 2014
KX584000	*Serinus canaria f. domestica*	Slovenia	Type A	Zadravec *et al.* 2017
KC215387	*Patagioenas fasciata monilis*	USA	Type A	Girard *et al.* 2014
EU881911	*Columba livia*	Spain	Type A	Sansano-Maestre *et al.* 2009
KJ776741	*Falco tinnunculus*	Spain	Type A	Martínez-Díaz *et al.* 2015
KJ721785	*Columba livia*	China	Type A	Jiang *et al.* 2016
KM095108	*Accipiter nisus*	Czech Republic	Type A	Kunca *et al.* 2015
JN007005	*Melopsittacus undulatus*	Austria	Type A	Reinmann *et al.* 2012
KJ776739	*Accipiter nisus*	Spain	Type A	Martínez-Herrero *et al.* 2014
KF993704	*Asio otus*	Spain	Type A	Martínez-Herrero *et al.* 2014

KF993703	*Strix aluco*	Spain	Type A	Martínez-Díaz *et al.* 2015
KF993702	*Circus cyaneus*	Spain	Type A	Martínez-Díaz *et al.* 2015
KF993701	*Buteo buteo*	Spain	Type A	Martínez-Herrero *et al.* 2014
KF993700	*Accipiter gentilis*	Spain	Type A	Martínez-Herrero *et al.* 2014
KF993699	*Aquila pennata*	Spain	Type A	Martínez-Herrero *et al.* 2014
KF993698	*Tyto alba*	Spain	Type A	Martínez-Herrero *et al.* 2014
KF993697	*Falco tinnunculus*	Spain	Type A	Martínez-Herrero *et al.* 2014
KF993696	*Bubo bubo*	Spain	Type A	Martínez-Herrero *et al.* 2014
KF993695	*Melopsittacus undulatus*	Spain	Type A	Martínez-Herrero *et al.* 2014
KF993694	*Streptopelia decaocto*	Spain	Type A	Martínez-Herrero *et al.* 2014
JQ755283	not provided	not provided	Type A	Peters & Raidal unpubl.
JQ755282	not provided	not provided	Type A	Peters & Raidal unpubl.
JQ755281	not provided	not provided	Type A	Peters & Raidal unpubl.
JQ755280	not provided	not provided	Type A	Peters & Raidal unpubl.

MOLECULAR ECOLOGY

JQ755279	not provided	not provided	Type A	Peters & Raidal unpubl.
KF993693	*Pica pica*	Spain	Type A	Martínez-Díaz *et al.* 2015
KX459443	*Columba oenas*	Germany	Type A	Marx *et al.* 2017
MH733817	*Columba livia*	China	Type A	Feng *et al.* 2018
KX844991	*Streptopelia decaocto*	Malta	Type A	Marx *et al.* 2017
EU215368*	*Buteo platypterus*	USA	Type B	Gerhold *et al.* 2008
MN587099*	*Streptopelia turtur*	UK	Type C	This study
MN587100	*Streptopelia turtur*	France	Type C	This study
MN587101	*Streptopelia turtur*	Senegal	Type C	This study
EU215362	*Columba livia*	UK	Type C	Gerhold *et al.* 2008
KX459499	*Streptopelia turtur*	Spain	Type C	Marx *et al.* 2017
KX459505	*Streptopelia turtur*	Italy	Type C	Marx *et al.* 2017
KX459497	*Streptopelia turtur*	Spain	Type C	Marx *et al.* 2017
KX459493	*Streptopelia turtur*	Spain	Type C	Marx *et al.* 2017

KX459498	*Streptopelia turtur*	Spain	Type C	Marx *et al.* 2017
MH733822	*Columba livia*	China	Type C	Feng *et al.* 2018
MH733821	*Columba livia*	China	Type C	Feng *et al.* 2018
KX459490	*Streptopelia turtur*	Spain	Type C	Marx *et al.* 2017
KX459489	*Streptopelia turtur*	Spain	Type C	Marx *et al.* 2017
KX459477	*Columba palumbus*	Germany	Type C	Marx *et al.* 2017
KX459473	*Columba palumbus*	Germany	Type C	Marx *et al.* 2017
KX459472	*Columba palumbus*	Germany	Type C	Marx *et al.* 2017
KX459455	*Columba palumbus*	Germany	Type C	Marx *et al.* 2017
KX459470	*Columba palumbus*	Germany	Type C	Marx *et al.* 2017
KX459476	*Columba palumbus*	Germany	Type C	Marx *et al.* 2017
MH733820	*Columba livia*	China	Type C	Feng *et al.* 2018
MH733819	*Columba livia*	China	Type C	Feng *et al.* 2018
KX459475	*Columba palumbus*	Germany	Type C	Marx *et al.* 2017

KX459471	*Columba palumbus*	Germany	Type C	Marx *et al.* 2017
MH733816	*Columba livia*	China	Type C	Feng *et al.* 2018
KX459485	*Streptopelia turtur*	Spain	Type C	Marx *et al.* 2017
KX459474	*Columba palumbus*	Germany	Type C	Marx *et al.* 2017
MH733818	*Columba livia*	China	Type C	Feng *et al.* 2018
KX266926	unspecified Pigeon sp.	Iran	Type C	Rajabloo *et al*. unpubl.
KX266924	unspecified Pigeon sp.	Iran	Type C	Rajabloo *et al*. unpubl.
MK418258	*Columba livia*	Iraq	Type C	Fahdil & Faraj unpubl.
MK418257	*Columba livia*	Iraq	Type C	Fahdil & Faraj unpubl.
MK418243	*Columba livia*	Iraq	Type C	Fahdil & Faraj unpubl.
MK418242	*Columba livia*	Iraq	Type C	Fahdil & Faraj unpubl.
MK418241	*Columba livia*	Iraq	Type C	Fahdil & Faraj unpubl.
MK418239	*Columba livia*	Iraq	Type C	Fahdil & Faraj unpubl.
EU215362	*Columba livia*	USA	Type C	Gerhold *et al.* 2008

KT869156	*Columba livia*	Iran	Type C	Ghorbani *et al*. unpubl.
KT869154	*Falco tinnunculus*	Iran	Type C	Ghorbani *et al*. unpubl.
KT869153	*Spilopelia senegalensis*	Iran	Type C	Ghorbani *et al*. unpubl.
KT869151	*Melopsittacus undulatus*	Iran	Type C	Ghorbani *et al*. unpubl.
KT869150	*Columba livia*	Iran	Type C	Ghorbani *et al*. unpubl.
EU881912	*Columba livia*	Spain	Type C	Sansano-Maestre *et al.* 2009
MF497423	unspecified Pigeon sp.	Iran	Type C	Soltani *et al.* unpubl.
KJ721784	*Columba livia*	China	Type C	Jiang *et al.* 2016
U86614	unspecified Pigeon sp.	Unknown	Type C	Felleisen 1997
JQ755289	*Accipiter fasciatus*	Unknown	Type C	Peters & Raidal unpubl.
KM246600	*Streptopelia decaocto*	Spain	Type C	Martínez-Díaz *et al.* 2015
KM246599	*Buteo buteo*	Spain	Type C	Martínez-Díaz *et al.* 2015
KJ776743	*Streptopelia decaocto*	Spain	Type C	Martínez-Díaz *et al.* 2015
KJ776740	*Circus cyaneus*	Spain	Type C	Martínez-Herrero *et al.* 2014

KF993692	*Buteo buteo*	Spain	Type C	Martínez-Herrero *et al.* 2014
KF993691	*Accipiter gentilis*	Spain	Type C	Martínez-Herrero *et al.* 2014
KF993690	*Falco peregrinus*	Spain	Type C	Martínez-Herrero *et al.* 2014
KF993689	*Pernis apivoris*	Spain	Type C	Martínez-Herrero *et al.* 2014
KF993688	*Circus aeruginosus*	Spain	Type C	Martínez-Herrero *et al.* 2014
KF993687	*Asio otus*	Spain	Type C	Martínez-Herrero *et al.* 2014
KF993686	*Aquila pennata*	Spain	Type C	Martínez-Herrero *et al.* 2014
KF993685	*Falco naumanni*	Spain	Type C	Martínez-Herrero *et al.* 2014
KF993684	*Accipiter gentilis*	Spain	Type C	Martínez-Herrero *et al.* 2014
KF993683	*Falco tinnunculus*	Spain	Type C	Martínez-Herrero *et al.* 2014
KF993682	*Columba palumbus*	Spain	Type C	Martínez-Herrero *et al.* 2014
KF993681	*Columba palumbus*	Spain	Type C	Martínez-Herrero *et al.* 2014
KF993680	*Streptopelia decaocto*	Spain	Type C	Martínez-Herrero *et al.* 2014
KF993679	*Columba livia*	Spain	Type C	Martínez-Herrero *et al.* 2014

KX459513*	*Streptopelia turtur*	Italy	Type C/V/N	Marx *et al.* 2017
EU215359*	*Columbina passerina*	USA	Type G	Gerhold *et al.* 2008
EU215360	*Zenaida asiatica*	USA	Type H	Gerhold *et al.* 2008
EU215361*	*Zenaida asiatica*	USA	Type I	Gerhold *et al.* 2008
EU215365*	*Zenaida macroura*	USA	Type J	Gerhold *et al.* 2008
EU215367*	*Patagioenas fasciata*	USA	Type K	Gerhold *et al.* 2008
KX459483*	*Streptopelia turtur*	Spain	Type P	Marx *et al.* 2017
KX459510*	*Streptopelia turtur*	Italy	Type Q	Marx *et al.* 2017

±These lineages are both labelled as Lineage III in Marx et al 2017, but are not identical over the region we examine so are labelled a and b here.

MOLECULAR ECOLOGY

Appendix S3. Summary of available Fe-hyd sequences on GenBank (accessed 15/05/2019) and consolidated into strains for construction of Figure 2, using the search term '*Trichomonas gallinae* Fe hydrogenase or Fe-hydrogenase or iron hydrogenase') (n=72) and the six from this study.

**Accession number**	**Host**	**Location**	**Strain ID**	**Reference**
JF681137*	*Alectroenas pulcherrimus*	Seychelles	1	Lawson *et al.* 2011
JF681138*	*Alectroenas pulcherrimus*	Seychelles	4	Lawson *et al.* 2011
JF681139*	*Alectroenas pulcherrimus*	Seychelles	5	Lawson *et al.* 2011
JF681140*	*Alectroenas pulcherrimus*	Seychelles	7	Lawson *et al.* 2011
JF681142*	*Geopelia striata*	Seychelles	12	Lawson *et al.* 2011
MT418244*	*Streptopelia turtur*	France	A1.1	This study
MT418245	*Streptopelia turtur*	UK	A1.1	This study
KC529660	*Accipiter nisus*	UK	A1.1	Chi *et al.* 2013
KC962158*	*Columba palumbus*	UK	A1.2	Chi *et al.* 2013
JF681136	*Chloris chloris*	UK	A1.2	Lawson *et al.* 2011

KJ184169	*Spinus tristis*	Canada	A1.2	McBurney *et al.* 2015
KJ184167	*Spinus tristis*	Canada	A1.2	McBurney *et al.* 2015
KC244203	*Zenaida macroura*	USA	A1.2	Girard *et al.* 2014
KC244201	*Patagioenas fasciata monilis*	USA	A1.2	Girard *et al.* 2014
HG008114	*Chloris chloris*	Multiple	A1.2	Ganas *et al.* 2014
MK172848	*Chloris chloris*	France	A1.2	Chavatte *et al.* 2019
MK172849	*Carduelis carduelis*	France	A1.2	Chavatte *et al.* 2019
MK172850	*Chloris chloris*	France	A1.2	Chavatte *et al.* 2019
MK172851	*Chloris chloris*	France	A1.2	Chavatte *et al.* 2019
MK172852	*Chloris chloris*	France	A1.2	Chavatte *et al.* 2019
KP900027	*Falco tinnunculus*	Spain	A1.2	Sansano-Maestre *et al.* 2016
KX514380	*Bubo bubo*	Spain	A1.2	Martínez-Herrero *et al.* 2017
KP900023	*Accipiter gentilis*	Spain	A1.2	Sansano-Maestre *et al.* 2016
KX584002	*Serinus canaria f. domestica+*	Slovenia	A1.2	Zadravec *et al.* 2017

KP900024	*Aquila fasciata*	Spain	A1.2	Sansano-Maestre *et al.* 2016
KP900025	*Bubo bubo*	Spain	A1.2	Sansano-Maestre *et al.* 2016
KP900026	*Columba livia*	Spain	A1.2	Sansano-Maestre *et al.* 2016
KP900028	*Strix aluco*	Spain	A1.2	Sansano-Maestre *et al.* 2016
KP900029	*Tyto alba*	Spain	A1.2	Sansano-Maestre *et al.* 2016
KC529661*	*Columba palumbus*	UK	A1.3	Chi *et al.* 2013
JF681141*	*Nesoenas picturatus*	Madagascar	A2	Lawson *et al.* 2011
HG008115	*Melopsittacus undulatus+*	Austria	A2	Ganas *et al.* 2014
KJ184170	*Columba livia*	Canada	A2	McBurney *et al.* 2015
KC244200	*Patagioenas fasciata monilis*	USA	A2	Girard *et al.* 2014
KP900030	*Columba livia*	Spain	A2	Sansano-Maestre *et al.* 2016
KC660127±	*Patagioenas fasciata monilis*	USA	BTPI1	Girard *et al.* 2014
AF446077*	*Columba livia*	USA	C1	Voncken *et al.* 2002
KP900032*	*Aquila pennata*	Spain	C2.1	Sansano-Maestre *et al.* 2016

MOLECULAR ECOLOGY

KC529664	*Columba palumbus*	UK	C2.1	Chi *et al.* 2013
KP900031	*Streptopelia decaocto*	Spain	C2.1	Sansano-Maestre *et al.* 2016
KC529662*	*Columba palumbus*	UK	C4	Chi *et al.* 2013
KC249971	*Columba livia*	USA	C4	Girard *et al.* 2014
KP900035	*Accipiter gentilis*	Spain	C4	Sansano-Maestre *et al.* 2016
KP900036	*Aquila fasciata*	Spain	C4	Sansano-Maestre *et al.* 2016
KP900037	*Columba livia*	Spain	C4	Sansano-Maestre *et al.* 2016
KP900038	*Streptopelia decaocto*	Spain	C4	Sansano-Maestre *et al.* 2016
KY569261	*Columba livia*	UK	C4	Alrefaei *et al.* unpubl.
KX514374	*Columba livia*	Spain	C4	Martínez-Herrero *et al.* 2017
KJ184172	*Columba livia*	Canada	C4	McBurney *et al.* 2015
KP900039*	*Aquila fasciata*	Spain	C5	Sansano-Maestre *et al.* 2016
KP900040	*Columba livia*	Spain	C5	Sansano-Maestre *et al.* 2016
KP900041*	*Aquila pennata*	Spain	C6	Sansano-Maestre *et al.* 2016

MT418240*	*Streptopelia turtur*	France	C7	This study
KP900033	*Aquila pennata*	Spain	C7	Sansano-Maestre *et al.* 2016
KP900034	*Streptopelia decaocto*	Spain	C7	Sansano-Maestre *et al.* 2016
KY569256*	*Columba livia*	UK	C8	Alrefaei *et al.* unpubl.
MT418241*	*Streptopelia turtur*	France	C8-TD	This study
MT418242	*Streptopelia turtur*	Senegal	C8-TD	This study
MT418243	*Streptopelia turtur*	UK	C8-TD	This study
KY569258*	*Zenaida graysoni* +	UK	C10	Alrefaei *et al.* unpubl.
MT418239*	*Streptopelia turtur*	France	C11-TD	This study
KC529663	*Streptopelia decaocto*	UK	C11-TD/C3§	Chi *et al.* 2013
KY569260*	*Columba livia* and *Accipiter nisus*	UK	C12	Alrefaei *et al.* unpubl.
KJ184168*	*Carpodacus purpureus*	Canada	Fe-hyd2	McBurney *et al.* 2015
KJ184171*	*Columba livia*	Canada	Fe-hyd5	McBurney *et al.* 2015
KX894542*	*Columba livia domestica*	Iran	MR2	Rajabloo *et al*. unpubl.

KX894543*	*Columba livia domestica*	Iran	MR14	Rajabloo *et al*. unpubl.
KX894546*	*Columba livia domestica*	Iran	MR22	Rajabloo *et al*. unpubl.
KX894544	*Columba livia domestica*	Iran	MR22^	Rajabloo *et al*. unpubl.
KX894545	*Columba livia domestica*	Iran	MR22^	Rajabloo *et al*. unpubl.
KX894547	*Columba livia domestica*	Iran	MR22^	Rajabloo *et al*. unpubl.
KX894548	*Columba livia domestica*	Iran	MR22^	Rajabloo *et al*. unpubl.
KX894550	*Columba livia domestica*	Iran	MR22^	Rajabloo *et al*. unpubl.
KX894551*	*Columba livia domestica*	Iran	MR30	Rajabloo *et al*. unpubl.
KX894549	*Columba livia domestica*	Iran	MR30^	Rajabloo *et al*. unpubl.
KX894552*	*Columba livia domestica*	Iran	MR104	Rajabloo *et al*. unpubl.
MT418249*	*Streptopelia turtur*	France	T1-TD	This study
MT418250	*Streptopelia turtur*	Senegal	T1-TD	This study
MT418246*	*Streptopelia turtur*	France	T2-TD	This study
MT418247	*Streptopelia turtur*	Senegal	T2-TD	This study

MOLECULAR ECOLOGY

MT418248	*Streptopelia turtur*	UK	T2-TD	This study
KY675299	*Streptopelia turtur*	Malta	T2-TD	Marx *et al.* 2017
KY675297	*Streptopelia turtur*	Italy	T2-TD	Marx *et al.* 2017
KY675298	*Streptopelia turtur*	Italy	T2-TD	Marx *et al.* 2017

Sequences marked with a * are used as representative samples in the tree (all others are duplicates of these over the 591 bp length included in the tree. *Trichomonas vaginalis* (Accession number XM001310179) was included in the phylogeny as an outgroup.

± Sequence too short to include in alignment

§These two strains were identical over the 591bp alignment used to generate our phylogenetic tree, but differed elsewhere in the Fe-hyd region

+Likely captive bird

^Separate strain ID provided in Genbank but identical to specified strain at the relevant region, so single strain ID provided for clarity

MOLECULAR ECOLOGY

Appendix S4. Statistical analysis of co-infection likelihood for all strain pairs. Type A, GEO, Type C, Type III and Tcl-1 are strains of *T. gallinae*; HB-TD HC-TD, and HD-TD are *Haemoproteus* sp., and LA-TD, LB-TD, LD-TD, LE-TD, LG-TD and LJ-TD are *Leucocytozoon* sp.

**Strain 1**	**Strain 2**	**Strain 1 number infected**	**Strain 2 number infected**	**Observed number coinfected**	**Probability of co-occurrence**	**Expected number coinfected**	**P Lt**	**P Gt**
Type A	GEO	17	12	0	0.085	4.2	**0.002**	1.000
Type A	Type C	17	10	0	0.071	3.5	**0.008**	1.000
Type A	Type III	17	1	0	0.007	0.3	0.653	1.000
Type A	Tcl-1	17	1	0	0.007	0.3	0.653	1.000
Type A	HB-TD	17	22	7	0.156	7.6	0.469	0.752
Type A	HC-TD	17	12	2	0.085	4.2	0.121	0.974
Type A	LA-TD	17	5	4	0.035	1.7	0.996	**0.043**
Type A	LB-TD	17	11	2	0.078	3.8	0.173	0.958
Type A	HD-TD	17	1	1	0.007	0.3	1.000	0.347
Type A	LD-TD	17	1	1	0.007	0.3	1.000	0.347

MOLECULAR ECOLOGY

Type A	LE-TD	17	4	0	0.028	1.4	0.170	1.000
Type A	LG-TD	17	3	1	0.021	1.0	0.727	0.731
Type A	LJ-TD	17	1	0	0.007	0.3	0.653	1.000
GEO	Type C	12	10	0	0.050	2.4	**0.042**	1.000
GEO	Type III	12	1	0	0.005	0.2	0.755	1.000
GEO	Tcl-1	12	1	0	0.005	0.2	0.755	1.000
GEO	HB-TD	12	22	7	0.110	5.4	0.921	0.228
GEO	HC-TD	12	12	3	0.060	2.9	0.677	0.620
GEO	LA-TD	12	5	0	0.025	1.2	0.229	1.000
GEO	LB-TD	12	11	2	0.055	2.7	0.455	0.828
GEO	HD-TD	12	1	0	0.005	0.2	0.755	1.000
GEO	LD-TD	12	1	0	0.005	0.2	0.755	1.000
GEO	LE-TD	12	4	1	0.020	1.0	0.752	0.688
GEO	LG-TD	12	3	1	0.015	0.7	0.856	0.578

GEO	LJ-TD	12	1	1	0.005	0.2	1.000	0.245
Type C	Type III	10	1	0	0.004	0.2	0.796	1.000
Type C	Tcl-1	10	1	0	0.004	0.2	0.796	1.000
Type C	HB-TD	10	22	4	0.092	4.5	0.506	0.758
Type C	HC-TD	10	12	4	0.050	2.4	0.950	0.190
Type C	LA-TD	10	5	1	0.021	1.0	0.733	0.698
Type C	LB-TD	10	11	6	0.046	2.2	1.000	**0.005**
Type C	HD-TD	10	1	0	0.004	0.2	0.796	1.000
Type C	LD-TD	10	1	0	0.004	0.2	0.796	1.000
Type C	LE-TD	10	4	3	0.017	0.8	0.999	**0.023**
Type C	LG-TD	10	3	1	0.012	0.6	0.898	0.504
Type C	LJ-TD	10	1	0	0.004	0.2	0.796	1.000
Type III	Tcl-1	1	1	0	0.000	0.0	0.980	1.000
Type III	HB-TD	1	22	1	0.009	0.4	1.000	0.449

Type III	HC-TD	1	12	0	0.005	0.2	0.755	1.000
Type III	LA-TD	1	5	0	0.002	0.1	0.898	1.000
Type III	LB-TD	1	11	0	0.005	0.2	0.776	1.000
Type III	HD-TD	1	1	0	0.000	0.0	0.980	1.000
Type III	LD-TD	1	1	0	0.000	0.0	0.980	1.000
Type III	LE-TD	1	4	0	0.002	0.1	0.918	1.000
Type III	LG-TD	1	3	0	0.001	0.1	0.939	1.000
Type III	LJ-TD	1	1	0	0.000	0.0	0.980	1.000
Tcl-1	HB-TD	1	22	1	0.009	0.4	1.000	0.449
Tcl-1	HC-TD	1	12	0	0.005	0.2	0.755	1.000
Tcl-1	LA-TD	1	5	0	0.002	0.1	0.898	1.000
Tcl-1	LB-TD	1	11	0	0.005	0.2	0.776	1.000
Tcl-1	HD-TD	1	1	0	0.000	0.0	0.978	1.000
Tcl-1	LD-TD	1	1	0	0.000	0.0	0.978	1.000

Tcl-1	LE-TD	1	4	0	0.002	0.1	0.918	1.000
Tcl-1	LG-TD	1	3	0	0.001	0.1	0.939	1.000
Tcl-1	LJ-TD	1	1	0	0.000	0.0	0.978	1.000
HB-TD	HC-TD	22	12	6	0.110	5.4	0.772	0.468
HB-TD	LA-TD	22	5	2	0.046	2.2	0.599	0.755
HB-TD	LB-TD	22	11	7	0.101	4.9	0.961	0.141
HB-TD	HD-TD	22	1	1	0.009	0.4	1.000	0.449
HB-TD	LD-TD	22	1	1	0.009	0.4	1.000	0.449
HB-TD	LE-TD	22	4	2	0.037	1.8	0.769	0.613
HB-TD	LG-TD	22	3	1	0.027	1.3	0.578	0.841
HB-TD	LJ-TD	22	1	0	0.009	0.4	0.551	1.000
HC-TD	LA-TD	12	5	1	0.025	1.2	0.644	0.771
HC-TD	LB-TD	12	11	3	0.055	2.7	0.746	0.545
HC-TD	HD-TD	12	1	0	0.005	0.2	0.755	1.000

HC-TD	LD-TD	12	1	0	0.005	0.2	0.755	1.000
HC-TD	LE-TD	12	4	0	0.020	1.0	0.311	1.000
HC-TD	LG-TD	12	3	1	0.015	0.7	0.856	0.578
HC-TD	LJ-TD	12	1	0	0.005	0.2	0.755	1.000
LA-TD	LB-TD	5	11	1	0.023	1.1	0.689	0.737
LA-TD	HD-TD	5	1	1	0.002	0.1	1.000	0.102
LA-TD	LD-TD	5	1	1	0.002	0.1	1.000	0.102
LA-TD	LE-TD	5	4	1	0.008	0.4	0.953	0.359
LA-TD	LG-TD	5	3	0	0.006	0.3	0.719	1.000
LA-TD	LJ-TD	5	1	0	0.002	0.1	0.898	1.000
LB-TD	HD-TD	11	1	0	0.005	0.2	0.776	1.000
LB-TD	LD-TD	11	1	0	0.005	0.2	0.776	1.000
LB-TD	LE-TD	11	4	4	0.018	0.9	1.000	**0.002**
LB-TD	LG-TD	11	3	0	0.014	0.7	0.458	1.000

LB-TD	LJ-TD	11	1	1	0.005	0.2	1.000	0.224
HD-TD	LD-TD	1	1	1	0.000	0.0	1.000	**0.020**
HD-TD	LE-TD	1	4	0	0.002	0.1	0.918	1.000
HD-TD	LG-TD	1	3	0	0.001	0.1	0.939	1.000
HD-TD	LJ-TD	1	1	0	0.000	0.0	0.980	1.000
LD-TD	LE-TD	1	4	0	0.002	0.1	0.918	1.000
LD-TD	LG-TD	1	3	0	0.001	0.1	0.939	1.000
LD-TD	LJ-TD	1	1	0	0.000	0.0	0.980	1.000
LE-TD	LG-TD	4	3	0	0.005	0.2	0.770	1.000
LE-TD	LJ-TD	4	1	0	0.002	0.1	0.918	1.000
LG-TD	LJ-TD	3	1	0	0.001	0.1	0.939	1.000

Table shows the number of birds infected with either strain for each pairwise comparison, and the number of birds co-infected with both, along with the probability of co-occurrence based on the occurrence of each strain within the population and expected number of co-infections. P Lt and P Gt represent the probabilities that these species could co-occur less (P Lt) or more often (P Gt) than observed in our data, respectively. Significant deviations from random (where $p<0.05$) are highlighted in bold

MOLECULAR ECOLOGY

Appendix S5. Full model results for General Linear Models (GLMs) testing for an effect of a) the number of parasite strains and b) the presence of coinfecting parasites, on body condition (weight). All estimates are from scaled variables.

a)	Estimate	SE	t	p
Intercept	<0.001	0.150	0.001	1.000
Wing length	0.177	0.153	1.153	0.256
Time of day (hour)	-0.125	0.153	-0.817	0.418
Number of strains	-0.085	0.151	-0.558	0.580

b)	Estimate	SE	t	p
Intercept	0.050	0.467	0.108	0.914
Wing length	0.179	0.154	1.162	0.252
Time of day (hour)	-0.129	0.160	-0.803	0.427
Coinfection	-0.057	0.498	-0.114	0.910

References

Chavatte J, Giraud P, Esperet D *et al.* (2019) An outbreak of trichomonosis in European greenfinches *Chloris chloris* and European goldfinches *Carduelis carduelis* wintering in Northern France. *Parasite*, **26**, 21.

Chi J, Lawson B, Durrant C *et al.* (2013) The finch epidemic strain of *Trichomonas gallinae* is predominant in British non-passerines. *Parasitology*, **140**, 1234–1245.

Felleisen R (1997) Comparative sequence analysis of 5 center dot 8S rRNA genes and internal transcribed spacer (ITS) regions of trichomonadid protozoa. *Parasitology*, **115**, 111–119.

Feng S, Chang H, Li F *et al.* (2018) Prevalence and molecular characterization of *Trichomonas gallinae* from domestic pigeons in Beijing, China. *Infection, Genetics and Evolution*, **65**, 369–372.

Ganas P, Jaskulska B, Lawson B *et al.* (2014) Multi-locus sequence typing confirms the clonality of *Trichomonas gallinae* isolates circulating in European finches. *Parasitology*, **141**, 652–661.

Gerhold R, Yabsley M, Smith A *et al.* (2008) Molecular characterization of the *Trichomonas gallinae* morphologic complex in the United States. *Journal of Parasitology*, **94**, 1335–1341.

Girard Y, Rogers K, Woods L *et al.* (2014) Dual-pathogen etiology of avian trichomonosis in a declining band-tailed pigeon population. *Infection, Genetics and Evolution*, **24**, 146–156.

Grabensteiner E, Bilic I, Kolbe T, Hess M (2010) Molecular analysis of clonal trichomonad isolates indicate the existence of heterogenic species present in different birds and within the same host. *Veterinary Parasitology*, **172**, 53–64.

Jiang X, Sun J, Wang F, Li H, Zhao X (2016) Prevalence of *Trichomonas* spp. in domestic pigeons in Shandong Province, China, and genotyping by restriction fragment length polymorphism. *Veterinary Journal*, **211**, 88–93.

Kunca T, Smejkalová P, Čepička I (2015) Trichomonosis in Eurasian sparrowhawks in the Czech Republic. *Folia Parasitologica*, **62**, 035.

Lawson B, Cunningham A, Chantrey J *et al.* (2011) A clonal strain of *Trichomonas gallinae* is the aetiologic agent of an emerging avian

epidemic disease. *Infection, Genetics and Evolution*, **11**, 1638–1645.

Martínez-Díaz R, Ponce-Gordo F, Rodríguez-Arce I *et al.* (2015) *Trichomonas gypaetinii* n. sp., a new trichomonad from the upper gastrointestinal tract of scavenging birds of prey. *Parasitology Research*, **114**, 101–112.

Martínez-Herrero M, Garijo-Toledo M, Liebhart D *et al.* (2017) Novel avian oropharyngeal trichomonads isolated from European turtle doves (*Streptopelia turtur*) and racing pigeons (*Columba livia*): genetic and morphometric characterisation of clonal cultures. *Infection, Genetics and Evolution*, **55**, 93–103.

Martínez-Herrero M, Sansano-Maestre J, López Márquez I *et al.* (2014) Genetic characterization of oropharyngeal trichomonad isolates from wild birds indicates that genotype is associated with host species, diet and virulence. *Avian Pathology*, **43**, 1–39.

Marx M, Reiner G, Willems H *et al.* (2017) High prevalence of *Trichomonas gallinae* in wild columbids across western and southern Europe. *Parasites and Vectors*, **10**.

McBurney S, Kelly-Clark W, Forzán M *et al.* (2015) Molecular characterization of *Trichomonas gallinae* isolates recovered from the Canadian Maritime provinces' wild avifauna reveals the presence of the genotype responsible for the European finch trichomonosis epidemic and additional strains. *Parasitology*, **142**, 1053–1062.

Nicholls J, Double M, Rowell D, Magrath R (2000) The evolution of cooperative and pair breeding in thornbills. *Journal of Avian Biology*, **2**, 165–176.

Reinmann K, Müller N, Kuhnert P *et al.* (2012) *Tritrichomonas foetus* isolates from cats and cattle show minor genetic differences in unrelated loci ITS-2 and EF-1α. *Veterinary Parasitology*, **185**, 138–44.

Robinson R, Lawson B, Toms M *et al.* (2010) Emerging infectious disease leads to rapid population declines of common British birds. *PLoS ONE*, **5**, e12215.

Sansano-Maestre J, Garijo-Toledo M, Gómez-Muñoz M (2009) Prevalence and genotyping of *Trichomonas gallinae* in pigeons and birds of prey. *Avian Pathology*, **38**, 201–207.

Sansano-Maestre J, Martínez-Herrero M, Garijo-Toledo M, Gómez-Muñoz M (2016) RAPD analysis and sequencing of ITS1/5.8S rRNA/ITS2

and Fe-hydrogenase as tools for genetic classification of potentially pathogenic isolates of *Trichomonas gallinae*. *Research in Veterinary Science*, **107**, 182–189.

Voncken F, Boxma B, van Hoek A *et al.* (2002) A hydrogenosomal [Fe]-hydrogenase from the anaerobic chytrid *Neocallimastix* sp. L2. *Gene*, **284**, 103–112.

Zadravec M, Slavec B, Krapež U *et al.* (2017) Trichomonosis outbreak in a flock of canaries (*Serinus canaria f. domestica*) caused by a finch epidemic strain of *Trichomonas gallinae*. *Veterinary Parasitology*, **239**, 90–93.
